# A systematic review of the psychosocial factors associated with pain in children with juvenile idiopathic arthritis

**DOI:** 10.1186/s12969-023-00828-5

**Published:** 2023-06-16

**Authors:** Yvonne N. Brandelli, Christine T. Chambers, Sean P. Mackinnon, Jennifer A. Parker, Adam M. Huber, Jennifer N. Stinson, Emily M. Wildeboer, Jennifer P. Wilson, Olivia Piccolo

**Affiliations:** 1 Department of Psychology and Neuroscience, Dalhousie University, Halifax, NS Canada; 2 Centre for Pediatric Pain Research, IWK Health Centre, 5859/5980 University Avenue, PO BOX 9700, Halifax, NS B3K 6R8 Canada; 3 Department of Pediatrics, Dalhousie University, Halifax, NS Canada; 4 Division of Pediatric Rheumatology, IWK Health, Halifax, NS Canada; 5 Lawrence S. Bloomberg Faculty of Nursing, University of Toronto, Toronto, ON Canada; 6 Research Institute, Hospital for Sick Children, Toronto, ON Canada; 7 Cassie and Friends: A Society for Children with Juvenile Arthritis and Other Rheumatic Diseases, Vancouver, BC Canada

**Keywords:** Systemic Review, Juvenile Idiopathic Arthritis, Pain, Correlates, Prognostic Factors, Mental Health, Quality of Life, Parent, Psychosocial Factors

## Abstract

**Background:**

Pain is one of the most frequently reported experiences amongst children with Juvenile Idiopathic Arthritis (JIA); however, the management of JIA pain remains challenging. As pain is a multidimensional experience that is influenced by biological, psychological, and social factors, the key to effective pain management lies in understanding these complex relationships. The objective of this study is to systematically review the literature on psychosocial factors of children with JIA and their caregivers 1) associated with and 2) predictive of later JIA pain intensity, frequency, and sensitivity in children 0–17 years of age.

**Methods:**

The Joanna Briggs Institute methodology for etiology and risk and Preferred Reporting Items for Systematic Reviews and Meta-analysis (PRISMA) statement guided the conduct and reporting of this review. Terms related to pain and JIA were searched in English without date restrictions across various databases (PubMed, CINAHL, PsycINFO, Embase, Scopus, and the Cochrane Central Register of Controlled Trials) in September 2021. Two independent reviewers identified, extracted data from, and critically appraised the included studies. Conflicts were resolved via consensus.

**Results:**

Of the 9,929 unique studies identified, 61 were included in this review and reported on 516 associations. Results were heterogeneous, likely due to methodological differences and moderate study quality. Results identified predominantly significant associations between pain and primary and secondary appraisals (e.g., more child pain beliefs, lower parent/child self-efficacy, lower child social functioning), parent/child internalizing symptoms, and lower child well-being and health-related quality of life. Prognostically, studies had 1-to-60-month follow-up periods. Fewer beliefs of harm, disability, and no control were associated with lower pain at follow-up, whereas internalizing symptoms and lower well-being were predictive of higher pain at follow-up (bidirectional relationships were also identified).

**Conclusions:**

Despite the heterogeneous results, this review highlights important associations between psychosocial factors and JIA pain. Clinically, this information supports an interdisciplinary approach to pain management, informs the role of psychosocial supports, and provides information to better optimize JIA pain assessments and interventions. It also identifies a need for high quality studies with larger samples and more complex and longitudinal analyses to understand factors that impact the pain experience in children with JIA.

**Trial registration:**

PROSPERO CRD42021266716.

**Supplementary Information:**

The online version contains supplementary material available at 10.1186/s12969-023-00828-5.

Pain is a common experience reported by children with Juvenile Idiopathic Arthritis (JIA) [1]. The pain is variable in intensity [2, 3], enduring [4, 5], only mildly associated with disease activity [6, 7], and associated with a host of negative outcomes (e.g., reduced participation, quality of life, and mental health challenges; e.g., [8–10]). In a recent qualitative study, healthcare providers (HCP) identified a lack of training and confidence in managing JIA pain, which led some to actively avoid talking about pain [11]. Evidently, there are important unmet needs pertaining to the understanding, assessment, and management of pain in JIA [12].

Pain is defined as “an unpleasant sensory and emotional experience associated with, or resembling that associated with, actual or potential tissue damage […] that is a personal experience that is influenced to varying degrees by biological, psychological, and social factors” [13]. In other words, pain is developed and maintained by biological (e.g., genetics, disease activity, medications), psychological (e.g., emotions, cognitions), and social/environmental (e.g., parents, peers) factors. Thus, while biological factors such as a diagnosis of JIA can increase one’s susceptibility and sensitivity to noxious stimuli, psychological and social (i.e., psychosocial) factors can also influence how pain is perceived. This is particularly important in the context of pediatric pain, wherein parent and family factors can interact with a child’s development to affect their pain experience [14]. In considering the transactional model of stress and coping [15], while the presence of JIA pain may present as a potential stressor, primary appraisals (e.g., whether it is perceived as dangerous), secondary appraisals (e.g., whether an individual has sufficient internal and external resources to manage it), how one copes, and its subsequent outcomes (e.g., well-being, mental health) can all influence the pain experience. Understanding the components that develop and maintain one’s pain are crucial to advancing the knowledge and management of JIA pain.

The relationships between biological, psychological, and social factors and JIA pain have been explored to varying degrees over the past four decades. Biological and disease-related factors have been explored extensively. Worse pain has been associated with enthesitis-subtype [16], greater active joint count [16], greater functional impairment [4], and greater sleep disturbance [17], whereas engagement in physical activity has been shown to be associated with decreased pain [18–21]. Age and sex have more inconsistent results [22], although recent research has suggested that females are at slightly greater risk of worse pain [23]. Psychosocial factors have been explored to a lesser degree. While the child’s mood/mental health [8], quality of life/well-being (e.g., [24]), cognitions and coping strategies (e.g., [25]), family functioning (e.g., [26]), and psychological therapies [27, 28] have also been explored in relation to JIA pain, results across these variables are not always consistent and have been measured in different ways.

The sensation of pain, for example, can be measured in terms of its intensity, frequency, or sensitivity in response to a noxious stimuli (i.e., hyperalgesia). Even these measures can be assessed in different ways (e.g., paper or electronic diaries, current or retrospective reports, self- or proxy-reports [29]), all of which can affect the interpretation and comparability of results. As such, a formalized review is needed to make sense of discrepancies across studies and accurately interpret findings in the context of methodological differences. Moreover, the synthesis of details such as study sample size, age, diagnosis, measures, and research design (e.g., whether factors are correlated or predictive) allows readers to fully ascertain the landscape of information.

Given the greater emphasis and consistency in the literature about what biological and disease-related factors are most relevant to consider, the emphasis of this review is on psychosocial factors. The objective of this study is to synthesize the literature on factors associated with JIA-related pain to determine what psychosocial factors in both individuals with JIA and others in their environment (e.g., caregivers) are 1) associated with and 2) predictive of (i.e., prognostic factors) JIA pain (intensity, frequency, sensitivity).

## Methods

This systematic review followed the Joanna Briggs Institute (JBI) methodology for etiology and risk [30] and The Preferred Reporting Items for Systematic Reviews and Meta-analysis (PRISMA) [31]. This study was pre-registered with the international prospective register of systematic reviews (PROSPERO CRD42021266716).

### Eligibility criteria

#### Population

This review included studies about children (0–17 years of age) with a diagnosis of JIA. The cut-off age was 17 years as many youth transition from pediatric to adult health systems around that age [32]. Studies reporting on children with comorbidities or rheumatic diseases other than JIA [33] were excluded to avoid potential confounds. Studies including broader age ranges (e.g., 0–18 years of age) or diagnoses (e.g., juvenile rheumatic diseases) were retained only if data were reported separately for children ages 0–17 years with JIA. Self- and proxy-reported data were included.

#### Exposure and outcome

Studies were included if they explored psychosocial factors associated with pain. This review used the most frequently assessed sensory components of JIA pain as the outcome: pain intensity, frequency, and sensitivity. Psychosocial factors were defined as factors within oneself (e.g., beliefs, coping, mood/affect) and the environment (e.g., parent/family factors, school and social functioning) that were associated with pain [34]. Psychosocial factors were included with Aim 1 if they were associated with pain at any point in time (i.e., correlated with or predicted by pain) and in Aim 2 if they predicted later pain (i.e., temporal precedence was established).

#### Types of studies

All quantitative studies published in the English language were included. No date restrictions were applied; however, dates were considered in the synthesis of results given an important shift in the treatment of JIA in the 2000s with the advent of biological agents. Observational designs were considered associations, whereas cohort designs were considered prognostic depending on the analyses. Qualitative studies, studies not reporting original data (e.g., reviews), and the grey literature were excluded.

### Search strategy

The search strategy aimed to identify all published studies pertaining to this review. Following the JBI methodology, a three-step search strategy was applied with the support of an evidence synthesis librarian (LB). First, a limited search was conducted of PubMed, the Cumulative Index to Nursing and Allied Health Literature (CINAHL), and Medline at OVID with keywords related to JIA, pain [35], and pediatrics [36], to ensure the search strategy encompassed pertinent terms. Second, the comprehensive search, inclusive of any keywords and index terms identified in the limited search, was completed on September 21^st^, 2021 (Additional file 1). The databases searched included Medline at OVID, CINAHL, PsycINFO, the Cochrane Central Register of Controlled Trials, Embase, and Scopus. Third, the reference list (backwards) and citing articles (forwards) of the included articles were searched for any additional studies. The search was updated on June 7^th^, 2022 to identify any recently published articles.

### Study selection

References were uploaded to Covidence systematic review software (Veritas Health Innovation, Melbourne, Australia). Duplicates were removed automatically and manually. Titles and abstracts were double screened for eligibility by two independent reviewers (always YNB, either EMW or OP). Relevant full texts were located, uploaded, and double screened for eligibility by the same reviewers. Inter-rater agreement was established using a weighted Cohen’s Kappa (poor: κ < 0.00; slight: κ = 0.00 – 0.20, fair: κ = 0.21 – 0.40, moderate: κ = 0.41 – 0.60, substantial: κ = 0.61 – 0.80, and almost perfect: κ = 0.81 – 1.00) [37]. Discrepancies were resolved via consensus (YNB, EMW, and OP).

### Methodological quality assessment

The methodological quality of the included studies was critically appraised by two independent reviewers (always YNB, either EMW or OP) using the JBI critical appraisal instruments [30]. These standardized instruments assess the presence of various methodological limitations (e.g., participant selection, measurement bias, confounds) in a “yes”, “no”, or “unclear” format. Different instruments were used based on the study design and way in which the data relevant to this review were collected (i.e., separate instruments were used for analytical cross-sectional studies, cohort studies). No attempts were made to contact authors for additional information. Discrepancies were resolved via consensus (YNB, EMW, and OP).

### Data extraction

A data extraction template was developed and pilot tested for this review. The template included information regarding the study, population, measures, and results (Additional file 2). Two independent reviewers (always YNB, either EMW or OP) extracted data from the included articles and discrepancies were resolved through consensus (YNB, EMW, and OP).

### Data synthesis

Given the heterogeneity of associations explored, data were synthesized narratively and in tabular form. Studies were grouped together based on the psychosocial factors. Similarities (e.g., significance of associations) and differences (e.g., reporter) across studies were explored.

## Results

### Study inclusion

**Fig. 1:**
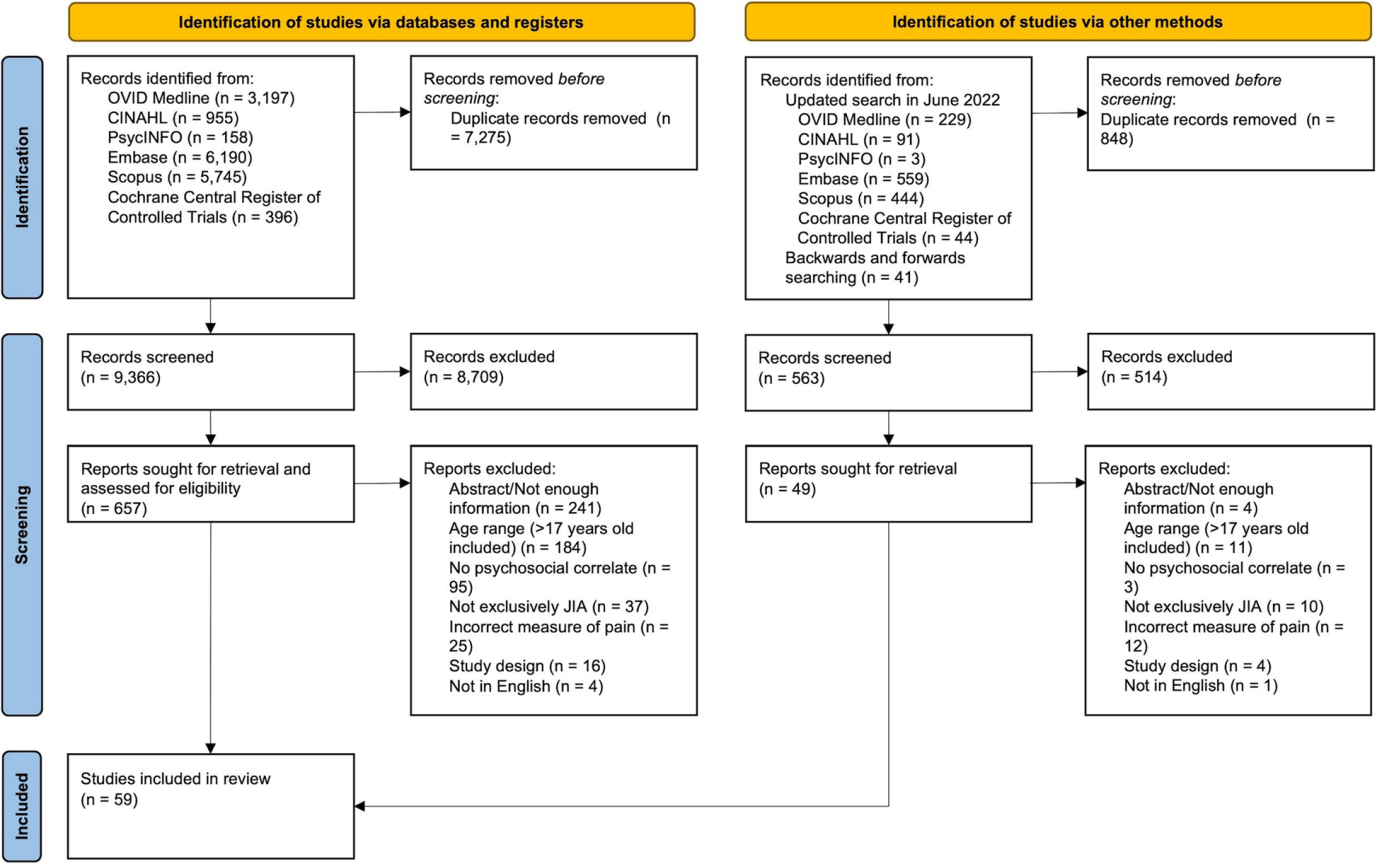
PRISMA chart detailing the search results and inclusion process

The systematic search returned 9,929 unique studies, 61 of which were included in this review [2–4, 25, 26, 38–91]. The PRISMA chart (Fig. 1) relays the search results and inclusion process [31]. Between rater reliability was moderate to substantial at the Title/Abstract screening stage (κ = 0.58 & 0.61) and substantial at the Full Text screening stage (κ = 0.61 & 0.73).

### Description of studies

The 61 included studies came from 59 articles and 49 unique datasets. Studies reporting on the same datasets were included only if new associations were identified (i.e., identical associations in multiple publications on the same dataset were removed). Publication dates ranged from 1987 to 2021. Most of the articles included were peer-reviewed publications, however two conference abstracts [66, 75] and six theses were also included [42, 46, 55, 57, 76, 84]. The six theses were selected over published manuscripts as additional associations were identified. Articles came from 17 countries, with the United States, Canada, the United Kingdom, and Denmark being the most represented. Most recruitment took place in clinics apart from two studies wherein it was unclear [59, 75]. Participants were predominantly children with JIA; however, 34 studies included parent/caregiver reports and two studies included HCP reports. Sample sizes ranged from 11 to 1906 participants (Mdn = 85; IQR = 99). Participants were largely female children (Mdn = 67%, IQR = 11%) and caregivers (Mdn = 83%, IQR = 17%), although some studies were missing these data. Other demographic information could not be aggregated given the variability of information reported on (e.g., medians or means, varying categories, missing information); however, most studies reported on children in the adolescent period (with only 7 studies including children younger than 5), with polyarticular and oligoarticular JIA as the most represented diagnoses.

Of the 516 unique associations, 234 were significant as per the α level used in each study. Fifty-one were classified as prognostic factors. Validated measures were generally used to measure pain intensity [65, 92–101]; although, 109 associations provided no or unclear references. Pain frequency [96, 97, 100, 102] and sensitivity [103, 104] were largely assessed using standardized measures and protocols. Pain was measured via self-report in 46 studies, proxy-report in 15 studies, and an unclear reporter in seven studies. Psychosocial factors were organized based on the transactional model of stress and coping [15] and included both child and parent factors. Validated measures were used to assess children’s primary appraisals (i.e., interpretations of whether JIA pain is positive, irrelevant, or threatening/harmful) [95, 99, 105]; children’s internal [44, 94, 106–109] and external [61, 63, 77, 78, 90, 93, 98, 106–108, 110–117] and parent’s internal [43, 108] secondary appraisals (i.e., assessment of resources available to manage JIA pain); children’s coping [82, 118–120]; and outcomes including children’s [94, 95, 99, 106–108, 110–112, 114, 121–136] and parent’s [108, 137–142] mental health, and children’s health-related quality of life (HRQOL; i.e., the impact of one’s health on their life [143]) and well-being (i.e., one’s sense of how well their needs are being met [144]) [98, 108, 110, 111, 143, 145–147]. Twenty associations exploring well-being provided no citation. Table 1 outlines the exact measures and their frequency of use. Six quasi-experimental studies explored pain in relation to psychosocial interventions [61, 63, 77, 78, 90]. The manipulation set them aside from other studies included in this review, thus the results have been included in Additional files 3.1–3.3 and the figures.

**Table 1:** Measures used in the 61 included studies (*N* = 516 associations)

Domain	Construct	Measure	Acronym	Frequency
Pain	Intensity	Pediatric Pain Questionnaire [100]	PPQ	168
		E-Ouch [99]	–	44
		Faces Pain Scale & Faces Pain Scale Revised [92, 97]	FPS(-R)	43
		Childhood Health Assessment Questionnaire [98]	CHAQ	40
		Patient-Reported Outcomes Measurement Information System [94]	PROMIS	9
		Recalled Pain Inventory [95, 99]	RPI	4
		Graded Chronic Pain Scale	GCPS	3
		Pain Intensity Scale [96]	PIS	3
		Child Health Assessment Questionnaire [93]	HAQ	2
		SUPERKIDZ [65]	–	2
		Juvenile Arthritis Multidimensional Assessment Report [101]	JAMAR	1
		No reference	–	109
	Frequency	Structured Pain Questionnaire [102]	SPQ	29
		Faces Pain Scale & Faces Pain Scale Revised [92, 97]	FPS(-R)	7
		Pain Intensity Scale [96]	PIS	3
		Pediatric Pain Questionnaire [100]	PPQ	4
		No reference	–	4
	Sensitivity	Quantitative Sensory Testing [103]	QST	24
		The Cold Pressor Task [104]	CPT	16
		No reference	–	1
Primary Appraisals	Pain Unpleasantness	E-Ouch [99]	–	3
		Recalled Pain Inventory [95, 99]	RPI	2
	Pain Beliefs	Survey of Pain Attitudes [105]	SOPA	28
Secondary Appraisals—Internal	Self-Efficacy	Children’s Arthritis Self-Efficacy Scale [44]	CASE	9
	Self-Esteem	Self-Perception Profile for Children and Adolescents [106, 107]	SPPC/A	3
		Child Health Questionnaire [108]	CHQ	2
	Stress	Patient-Reported Outcomes Measurement Information System [94]	PROMIS	2
		Perceived Stress Scale [109]	PSS-10	1
		No reference	–	1
	Physical Appearance	Self-Perception Profile for Children and Adolescents [106, 107]	SPPC/A	3
	Cognitive Function	Patient-Reported Outcomes Measurement Information System [94]	PROMIS	2
Parent Secondary Appraisals—Internal	Self-Efficacy	Parent Arthritis Self-Efficacy Scale [43]	PASE	16
	Self-Esteem	Child Health Questionnaire [108]	CHQ	1
Secondary Appraisals -External	School Functioning	Pediatric Quality of Life Inventory – Core & Arthritis Modules [110, 111]	PedsQL	8
		Childhood Health Assessment Questionnaire [98]	CHAQ	3
		Self-Perception Profile for Children and Adolescents [106, 107]	SPPC/A	3
		Child Health Assessment Questionnaire [93]	HAQ	2
		Revised Children’s Manifest Anxiety Scale [112]	RCMAS	1
		No reference	–	3
	Social Functioning	Social Skills Rating System [113]	SSRS	15
		Pediatric Quality of Life Inventory – Core & Arthritis Modules [110, 111]	PedsQL	10
		Child Behavior Checklist [114]	CBCL	3
		Self-Perception Profile for Children and Adolescents [106, 107]	SPPC/A	3
		Revised Children’s Manifest Anxiety Scale [112]	RCMAS	2
		Social Support Questionnaire – Revised [115]	SSQR	2
	Parent Pain Responses	West Haven-Yale Multidimensional Pain Inventory [116]	WHYMPI	9
	Family Relationships	Family Environment Scale [117]	FES	35
		Child Health Questionnaire [108]	CHQ	4
		No citation	–	2
	Interventions^a^	Pain Management Intervention [61]	–	9
		Cognitive Behavioral Therapy Intervention [90]	CBT	1
		Cognitive Behavioral Therapy Group Intervention [63]	CBT	10
		Peer-Led Intervention [77]	iPeer2Peer	1
		Self-Management Intervention [78]	TTC	3
Coping	Coping	Pain Coping Questionnaire^b^ [82]	PCQ	76
		Pain Catastrophizing Scale for Children [118]	PCS-C	9
		Coping Strategies Questionnaire for Children [119, 120]	CSQ-C	1
		No reference	–	2
Outcomes	General Mental Health	Pediatric Symptom Checklist [121]	PSC	8
		Child Behavior Checklist [114]	CBCL	5
		Child Health Questionnaire [108]	CHQ	2
		Rutter Parental Screening Questionnaire [148]	–	1
	Externalizing Symptoms	Child Behavior Checklist [114]	CBCL	5
		Child Health Questionnaire [108]	CHQ	4
		Self-Perception Profile for Children and Adolescents [106, 107]	SPPC/A	3
	Internalizing Symptoms	Pediatric Quality of Life Inventory – Core & Arthritis Modules [110, 111]	PedsQL	8
		Child Behavior Checklist [114]	CBCL	3
		Child Vulnerability Scale [122]	CVS	1
		Patient Health Questionnaire [123]	PHQ-4	1
		No reference	–	4
	Anxiety Symptoms	State-Trait Anxiety Inventory for Children [124]	STAI-C	12
		Revised Children’s Manifest Anxiety Scale [112]	RCMAS	4
		Pediatric Quality of Life Inventory – Core & Arthritis Modules [110, 111]	PedsQL	2
		Patient-Reported Outcomes Measurement Information System [94]	PROMIS	2
		Trauma Symptom Checklist for Children [125]	TSC-C	2
		Screen for Child Anxiety Related Disorders [126]	SCARED	1
	Mood/Depression Symptoms	Children’s Depression Inventory [127]	CDI	12
		Mood and Feelings Questionnaire [128]	MFQ	12
		Positive and Negative Affect Scale for Children [129]	PANAS-C	8
		Child Behavior Checklist [114]	CBCL	6
		Facial Affective Scale [130]	FAS	4
		Patient-Reported Outcomes Measurement Information System [94]	PROMIS	3
		Children’s Emotion Management Scale [131]	–	2
		Differential Emotions Scale – IV [132]	DES-IV	2
		Emotion Regulation Scale [133]	–	2
		Hopelessness Scale for Children [134]	–	2
		Revised Child Anxiety and Depression Scale [135]	RCADS	2
		Trauma Symptom Checklist for Children [125]	TSC-C	2
		Centre for Epidemiological Studies Depression Scale for Children [136]	CES-DC	1
	Pain Interference/ Limitations	Recalled Pain Inventory [95, 99]	RPI	6
		Child Health Questionnaire [108]	CHQ	4
		E-Ouch [99]	–	3
	Health-Related Quality of Life (HRQOL)	Pediatric Quality of Life Inventory – Core & Arthritis Modules [110, 111]	PedsQL	22
		Juvenile Arthritis Quality of Life Questionnaire [146]	JAQQ	7
		Child Health Questionnaire [108]	CHQ	5
		Quality of My Life Scale [143]	QoML	3
		Clinically Derived Global Score for Psychosocial Functioning [147]	CGAS	1
	Well-being	Global Assessment of Well-being Visual Analogue Scale	–	20
		Childhood Health Assessment Questionnaire [98]	CHAQ	7
		World Health Organization Well-Being Index [145]	WHO-5	1
Parent Outcomes	General Mental Health	Lanyon Psychological Screening Inventory [137]	–	2
		Self-Reporting Questionnaire [138]	SRQ-20	1
	Anxiety Symptoms	Hospital Anxiety and Depression Scale [139]	HADS	3
	Mood/ Depression Symptoms	Hospital Anxiety and Depression Scale [139]	HADS	3
		Beck Depression Inventory [140]	BDI	3
	Pain Interference/ Limitations	Revised Hassles and Uplifts Scale [141]	–	5
		Child Health Questionnaire [108]	CHQ	4
		Caregiver Burden Scale [142]	CBS	1

^a^ See Additional files 3 and 4 for results

^b^ Some studies used a preliminary version of this scale

### Methodological quality

The included studies were critically appraised using JBI tools [149, 150] based on the associations used in the review rather than the stated study design (e.g., daily diary studies were categorized as cross-sectional or cohort depending on how the data were analyzed, studies with pain predicting psychosocial factors were considered cross-sectional designs). For the two theses that contained two studies each, separate appraisals were conducted. Fifty-one studies were cross-sectional and five were cohort. No studies were excluded based on the critical appraisal.

The median critical appraisal score was 75% (IQR = 20%). For the 51 cross-sectional studies, scores ranged from 38 to 100%, with the identification and management of confounds as the greatest weakness (Table 2). For the five cohort studies, scores ranged from 40 to 89%, with the validity of the outcome measurement (i.e., pain) as the lowest rated item (Table 3).

**Table 2:** Critical appraisal results for analytical cross sectional studies

Author & Year	Q1	Q2	Q3	Q4	Q5	Q6	Q7	Q8	%
Amine 2009 [38]	Y	Y	U	Y	N	N	U	Y	50%
Anthony 2011^‡^ [39]	Y	Y	Y	Y	Y	Y	Y	Y	100%
Armbrust 2016 [40]	Y	Y	N	Y	Y	Y	U	Y	75%
Baildam 1995 [41]	Y	Y	Y	Y	N	N	Y	N	63%
Baloueff 1996 [42]	Y	Y	Y	Y	Y	Y	Y	Y	100%
Barlow 2000 [43]	Y	N	Y	Y	N	N	U	Y	50%
Barlow 2001 [44]	Y	N	Y	Y	N	N	U	Y	50%
Barlow 2002 [45]	N	N	Y	Y	N	N	Y	Y	50%
Bromberg 2009^‡^ [46]	Y	Y	Y	Y	N	N	Y	Y	75%
Bromberg 2012^‡^ [47]	Y	Y	Y	Y	Y	Y	Y	Y	100%
Bruns 2008 [48]	Y	Y	Y	Y	N	N	U	Y	63%
Cornelissen 2014 [50]	Y	Y	Y	Y	N	N	Y	Y	75%
Dimitrijevic Carlsson 2019 [51]	Y	Y	N	Y	N	N	Y	Y	63%
Doherty 1993 [52]	Y	Y	Y	Y	N	N	Y	Y	75%
El-Najjar 2014 [53]	Y	Y	Y	Y	N	N	U	Y	63%
Hagglund 1995 [54]	Y	Y	Y	Y	Y	Y	Y	Y	100%
Hanns 2018–2^‡‡^ [55]	Y	Y	Y	Y	Y	Y	U	Y	88%
Jaworski 1992 [57]	Y	N	Y	Y	N	N	Y	Y	63%
Klotsche 2014 [58]	Y	Y	U	Y	Y	Y	U	Y	75%
Kovalchuk 2017 [59]	N	N	U	Y	N	N	Y	Y	38%
Kovalchuk 2018 [60]	N	N	Y	Y	N	N	U	Y	38%
Listing 2018 [62]	Y	Y	Y	Y	U	U	U	Y	63%
Lomholt 2013^††^ [64]	Y	Y	Y	Y	N	N	Y	Y	75%
Luca 2017 [65]	Y	N	Y	Y	N	N	Y	Y	63%
Mahler 2017 [66]	Y	Y	U	Y	N	N	U	Y	50%
Margetić 2005 [67]	U	N	Y	Y	N	N	Y	Y	50%
Oen 2009^§^ [69]	Y	Y	U	Y	Y	Y	U	Y	75%
Oen 2021^§^ [68]	Y	N	U	Y	Y	Y	U	Y	63%
Ross 1993 [70]	Y	Y	Y	Y	Y	Y	Y	Y	100%
Sällfors 2004 [71]	Y	Y	Y	Y	N	N	N	Y	63%
Schanberg 2003^‡^ [2]	Y	Y	Y	Y	N	N	Y	Y	75%
Schanberg 2005^‡^ [72]	Y	Y	Y	Y	Y	Y	Y	Y	100%
Selvaag 2003 [73]	N	Y	N	Y	Y	Y	N	Y	63%
Selvaag 2005 [74]	N	N	Y	Y	N	N	U	Y	38%
Shelepina 2011 [75]	N	N	Y	U	N	N	U	Y	25%
Stinson 2006–1^†^ [76]	Y	Y	Y	Y	N	N	Y	Y	75%
Stinson 2006–2 [76]	Y	Y	Y	Y	N	N	Y	Y	75%
Tarakci 2011 [79]	Y	Y	Y	Y	N	N	Y	Y	75%
Tarkiainen 2019 [80]	Y	U	Y	Y	Y	Y	U	Y	75%
Thastum 1997 [81]	Y	Y	Y	Y	N	N	Y	Y	75%
Thastum 1998 [82]	N	N	Y	Y	N	N	Y	Y	50%
Thastum 2005^††^ [25]	Y	Y	Y	Y	Y	Y	Y	Y	100%
Thompson 1987 [26]	Y	U	Y	Y	N	N	Y	Y	63%
Tupper 2012 [84]	Y	Y	Y	Y	N	N	Y	Y	75%
Tupper 2013^†^ [3]	U	Y	Y	Y	Y	Y	Y	Y	88%
Upadhyay 2021 [85]	N	N	Y	Y	N	N	Y	Y	50%
Vandvik 1990 [86]	Y	N	Y	Y	N	N	Y	Y	63%
Vuorimaa 2008^§§^ [89]	Y	Y	Y	Y	N	N	U	U	50%
Vuorimaa 2009^§§^ [88]	Y	Y	Y	Y	N	N	U	U	50%
Vuorimaa 2011^§§^ [87]	Y	Y	Y	Y	N	N	Y	Y	75%
Yan 2020 [91]	Y	Y	Y	Y	N	N	Y	Y	75%
%	80%	69%	82%	98%	29%	29%	61%	94%	

JBI critical appraisal for quasi-experimental studies: Q1 = Were the criteria for inclusion in the sample clearly defined? Q2 = Were the study subjects and the setting described in detail? Q3 = Was the exposure measured in a valid and reliable way? Q4 = Were objective, standard criteria used for measurement of the condition? Q5 = Were confounding factors identified? Q6 = Were strategies to deal with confounding factors stated? Q7 = Were the outcomes measured in a valid and reliable way? Q8 = Was appropriate statistical analysis used?

*Y* Yes, *N* No, *U* Unclear

^‡, ‡‡, †, ††, §, §§^ Studies with overlapping datasets

**Table 3:** Critical appraisal results for cohort studies

Author & Year	Q1	Q2	Q3	Q4	Q5	Q6	Q7	Q8	Q9	Q10	Q11	%
Connelly 2012 [49]	N/A	N/A	Y	N	N	N	Y	Y	Y	Y	Y	67%
Hanns 2018–1^‡‡^ [55]	N/A	N/A	Y	Y	Y	N	U	Y	N	N	Y	56%
Hoff 2006 [56]	N/A	N/A	Y	Y	Y	N	Y	Y	Y	Y	Y	89%
Rashid 2018^‡‡^ [4]	U	N/A	U	Y	Y	N	U	Y	N	N	Y	40%
Thastum 2011^††^ [83]	N/A	N/A	Y	Y	Y	N	Y	Y	Y	Y	Y	89%
%	0%	N/A	80%	80%	80%	0%	60%	100%	60%	60%	100%	

JBI critical appraisal for cohort studies: Q1 = Were the two groups similar and recruited from the same population? Q2 = Were the exposures measured similarly to assign people to both exposed and unexposed groups? Q3 = Was the exposure measured in a valid and reliable way? Q4 = Were confounding factors identified? Q5 = Were strategies to deal with confounding factors stated? Q6 = Were the groups/participants free of the outcome at the start of the study (or at the moment of exposure)? Q7 = Were the outcomes measured in a valid and reliable way? Q8 = Was the follow up time reported and sufficient to be long enough for outcomes to occur? Q9 = Was follow up complete, and if not, were the reasons to loss to follow up described and explored? Q10 = Were strategies to address incomplete follow up utilized? Q11 = Was appropriate statistical analysis used?

*Y* Yes, *N* No, *U* Unclear, *N/A* Not applicable

^‡, ‡‡, ‡‡‡, †, ††, §, §§^ Studies with overlapping datasets

### Findings of the review

**Fig. 2:**
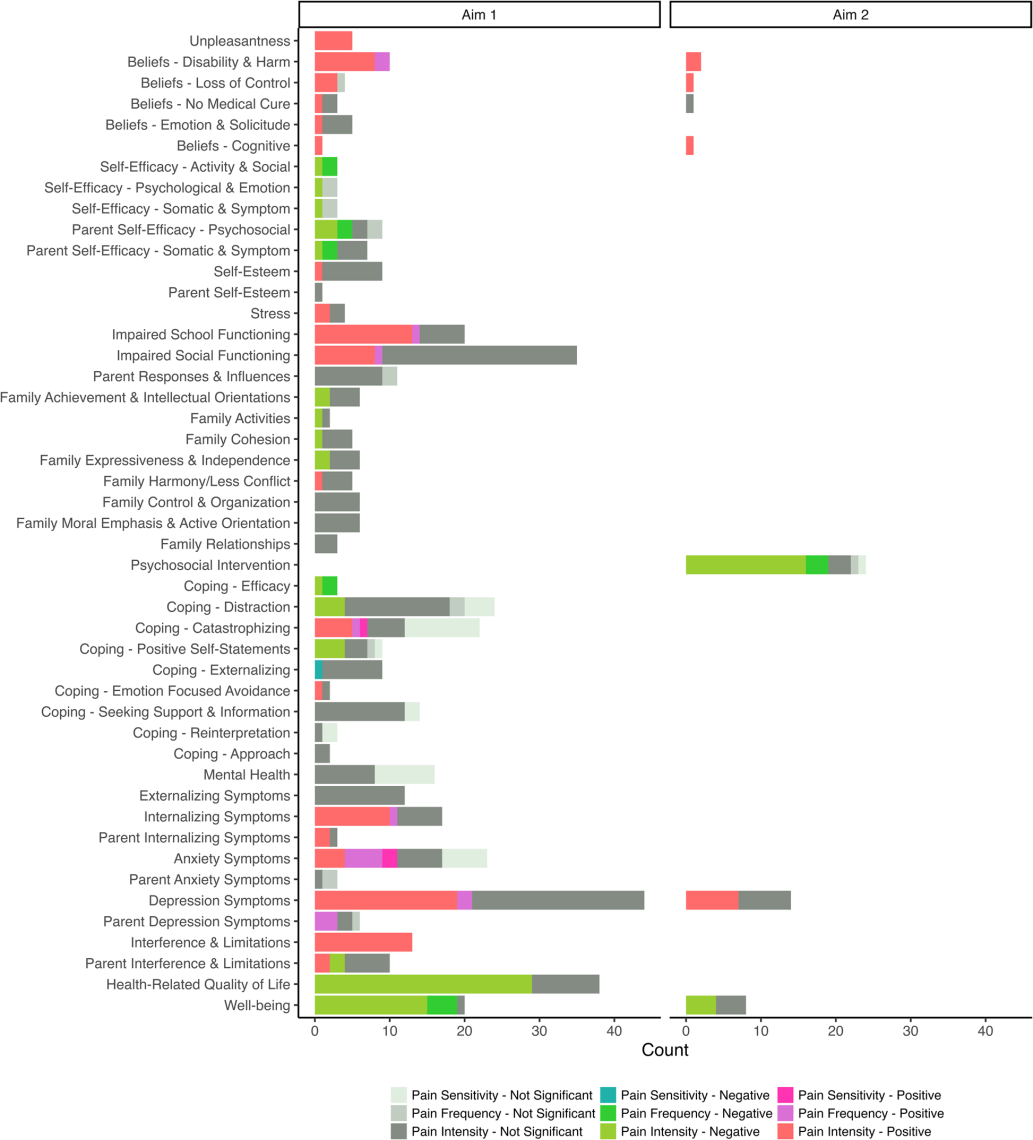
Psychosocial factors identified and their association with pain intensity, frequency, and sensitivity in youth with JIA

Findings of the review have been grouped based on the study aims, categories as they map to the transactional model of stress and coping [15], and child/parent factors. See Table 4 for study details and Fig. 2/Additional file 4 for a summary.

**Table 4:** Study characteristics and results

Author, Year, Publication Type	Sample Size(s)	Age(s) x̄ or Mdn (Range)	% Girls	% JIA Type	Pain: Construct (Reporter) – Measure	Psychosocial Factor(s): Construct (Reporter) – Measure	Main Findings: Analysis—Result
Amine 2009 [38] Article	80 C 80 P	x̄ = 11 (6–17) --	59 --	Po:32; O:43; S:26	• PI (--) – VAS CHAQ	• HRQOL (--) – JAQQ	• Corr – Lower well-being was significantly associated with greater PI
Anthony 2011^‡^ [39] Article	51 C 51 P	x̄ = 12 (8–16) --	61 96	Po:63; E:8; S:24; Ps:5	• PI (C) – VAS PPQ (current)	• P Depression symptoms (P) – BDI • Vulnerability (P) – CVS • P Hassles & Uplifts Intensity & Frequency (P) – Hassles and Uplifts Scale	• Corr – Parent depression symptoms, child vulnerability, parent identified daily hassles (intensity and frequency), and parent identified daily uplifts (intensity) were not significantly associated with PI • Corr & HR controlling for age, gender, active joint count, and disease severity – More parent reported daily uplifts were significantly associated with greater PI
Armbrust 2016 [40] Article	80 C	Mdn = 10 (8–13)	65	Po:35; O:45; E:4; S:11; Ps:5	• PI (C) – VAS	• School Attendance (C) – -- (yes/no)	• Corr & LoR controlling for age, disease activity, medications, disability, and fatigue – Lower school attendance was significantly associated with greater PI
Baildam 1995 [41] Article	29 C 29 P	x̄ = 11 (7–16) --	48 --	Po:48 O:52	• PI (C) – VAS (worst past week)	• Mental Health (P) – Rutter Parental Screening Questionnaire high (≥ 13) / low (< 13)	• Mann–Whitney U Test – Children with higher and lower Rutter scores did not significantly differ in PI
Baloueff 1996 [42] Thesis	60 C	x̄ = 12 (8–17)	73	Po:33; O:57; S:10	• PI (C) – VAS PPQ (average of current and past week) mean and high (> 2.5 cm)/ low (< 2.5 cm)	• Behavioral Conduct, Self-Esteem, Scholastic Competence, Appearance, & Social Acceptance (C) – SPPC/A • Assertion, Cooperation, Empathy, Self-Control, & Social Skills (C) – SSRS	• Corr, MR & one-way ANOVA – Behavioral conduct, physical appearance, scholastic competence, social acceptance, self-esteem, assertion, cooperation, empathy, self-control, and social skills were not significantly associated with PI, nor did they significantly differ between high and low pain groups
Barlow 2000 [43] Article	116 C 178 P	* (7–17) *	64 65	--	• PI (Mother, Father, & C) – VAS (current)	• P Psychosocial & Symptom Self-Efficacy (Mother & Father) – PASE	• Corr – Greater mother’s psychosocial self-efficacy was significantly associated with lower mother and child reports of PI Greater father’s psychosocial self-efficacy was significantly associated with lower PI as reported by the child but not themselves Greater mother’s symptom self-efficacy was significantly associated with lower PI as reported by themselves but not their child Father’s symptom self-efficacy was not significantly associated with their own and child reports of PI
Barlow 2001 [44] Article	89 C 151 P	x̄ = 12 (7–17) *	62 58	--	• PI (C) – VAS (current)	• Activity, Emotion, & Symptom Self-Efficacy (C) – CASE	• Corr – Greater child activity, emotion, and symptom self-efficacy were significantly associated with lower PI
Barlow 2002 [45] Article	30 C 30 P	x̄ = 11 (--) x̄ = 38 (--)	67 100	Po:26; O:61; S:13	• PI (C) – VAS PPQ (current)	• P Depression & Anxiety symptoms (Mother) – HADS • P Psychosocial & Symptom Self-Efficacy (Mother) – PASE	• Corr – Maternal depression and anxiety symptoms, and psychosocial and symptom self-efficacy were not significantly associated with PI
Bromberg 2009^‡^ [46] Thesis	51 C	x̄ = 12 (8–16)	65	Po:100	• PI (C) – VAS PPQ (1x/day for 2 mos)	• Coping Efficacy (C) – CSQ-C assessed 1x/day for 2 mos	• HR controlling for age, disease severity, and sleep quality – Greater coping efficacy was significantly associated with lower PI
Bromberg 2012^‡^ [47] Article	51 C 51 P	x̄ = 12 (8–16) --	65 --	Po:100	• PI (C) – VAS PPQ (1x/day for 2 mos)	• Mood (C) –VAS FAS assessed 1x/day for 2 mos	• Hierarchical MLM controlling for age, disease severity, and between and within child sleep quality – Higher daily reported mood (within subjects), but not mean mood (between subjects), was significantly associated with lower PI that day
Bruns 2008 [48] Article	70 C 70 P	x̄ = 10 (0–16) x̄ = 37 (--)	67 91	Po:63; O:16; S:21	• PI (--) – VAS (past week)	• P Caregiver Burden (P) – CBS • P Mental Health (P) – SRQ-20	• Corr – Caregiver burden and parent mental health were not significantly associated with PI
Connelly 2012 [49] Article	43 C 43 P	x̄ = 13 (8–17) --	86 90	--	• PI (C) – electronic VAS (3x/day for 28 days)	• Variability in positive & negative mood, ability to adaptively attenuate negative emotions, & ability to upregulate positive emotions (C) – PANAS-C assessed 3x/day for 28 days • Emotion Regulation (P) – The Emotion Regulation Scale (baseline) • Emotion Regulation (C) – Children’s Emotion Management Scale (baseline)	• Corr and LMM – Greater variability in positive and negative emotions were significantly associated with and predictive of greater PI A child’s ability to adaptively attenuate negative emotions was associated with, but not predictive of, lower PI A child’s ability to adaptively upregulate positive emotions to average levels following a drop was not significantly associated with but was predictive of lower PI Parent-reported and self-reported emotion regulation at baseline was not significantly associated with or predictive of PI
Cornelissen 2014 [50] Article	60 C	Mdn = 13 (7–17)	73	Po:48; Ps:22	• PS (C) – Cold Detection, Cold Pain, Warm Detection, Warm Pain, Mechanical Detection, Mechanical Pain, Vibration Detection, & Pressure Pain Thresholds	• Catastrophizing (C) – PCS-C • Mental Health (C) – PSC • Trait Anxiety symptoms (C) – STAI-C	• LR – Catastrophizing and mental health were not significantly associated with PS as measured by the child’s cold detection, cold pain, warm detection, heat pain, mechanical detection, mechanical pain, vibration detection, or pressure pain thresholds Greater trait anxiety symptoms were significantly associated with greater PS as measured by the child’s lower mechanical detection and mechanical pain thresholds, but not by their cold detection, cold pain, warm detection, heat pain, vibration detection, or pressure pain thresholds
Dimitrijevic Carlsson 2019 [51] Article	45 C	Mdn = 12 (6–16)	73	Po:33; O:44	• PI for temporo-mandibular joints (C) – GCPS (average of current, past week, and worst in the past week)	• Catastrophizing (C) – PCS-C • Distress (C) – PHQ-4 • Stress (C)—PSS	• Corr – Greater catastrophizing, distress, and perceived stress were significantly associated with greater temporomandibular joint PI
Doherty 1993 [52] Article	20 C 20 P	x̄ = 11 (8–15) --	55 100	Po:15; O:55; S:30	• PI (C & P) – VAS Child HAQ	• School absences (P) – Child HAQ	• Corr – More school absences were significantly associated with greater parent, but not child, reported PI
El-Najjar 2014 [53] Article	54 C 54 P	x̄ = 11 (6–15) --	67 --	Po:28; O:39; E:11; S:22	• PI (--) – VAS	• Depression symptoms (C) – CES-DC	• Corr – More depression symptoms were significantly associated with greater PI
Hagglund 1995 [54] Article	60 C	x̄ = 11 (7–17)	62	Po:35; O:55; S:10	• PI (C) – VAS (past month)	• Social Support (C) – SSQR • Hopelessness (C) – Hopelessness Scale for Children • Sadness (C) – DES-IV	• Corr and HR controlling for age, gender, socioeconomic status, disease duration, and articular severity – Social support, hopelessness, and sadness were not significantly associated with PI
Hanns 2018–1^‡‡^ [55] Thesis	219 C	x̄ = 13 (11–16)	57	Po:22; O:35; E:13; S:6; Ps:13; U:11	• PI (C) – VAS (baseline, 6, and 12 mos) mean and high (7.4)/low (0.4)	• Depression symptoms (C) – MFQ at baseline, 6, and 12 mos average and low (2 points)/high (31 points)	• LMM controlling for active/limited joint count and disability – More depression symptoms at baseline significantly predicted greater PI over time, and greater PI at baseline predicted more depression symptoms over time • Mann Whitney U-Test – More depression symptoms at baseline significantly predicted greater PI over 12 mos, and higher PI at baseline significantly predicted greater depression symptoms over 12 mos
Hanns 2018–2^‡‡^ [55] Thesis	102 C	Mdn = 13 (11–16)	57	Po:30; O:52; E:18	• PI (C) – VAS	• Depression symptoms (C) – MFQ mean and high (≥ 27)/low (< 27)	• Corr and MR controlling for age, medications, diagnosis, gender – Greater depression symptoms were significantly associated with greater PI • Mann Whitney U-Test – Children with high and low depression symptoms did not significantly differ in PI
Hoff 2006 [56] Article	63 C 63 P	x̄ = 12 (8–17) x̄ = 40 (--)	81 --	Po:29; O:41; E:8; S:5; U:18	• PI (C & P) – FPS (last few days at baseline, 6, and 12 mos)	• Depression symptoms (C) – RCADS at baseline	• LMM controlling for age, gender, income, and disease severity – Greater depressive symptoms at baseline significantly predicted child reported, but not parent reported, PI over time when PI was low at baseline
Jaworski 1992 [57] Thesis	30 C 30 P	x̄ = 11 (6–17) --	73 --	Po:73; O:27	• PI (C & P) – VAS PPQ	• Depression symptoms (C) – CDI • Depression symptoms (P) – CBCL • P Punishing, Distracting, & Solicitous Pain Responses (P) – WHYMPI	• Corr – Child reported depression symptoms were significantly associated with greater parent reported PI for the whole sample, and 12–17-year-olds, but not 6–11-year-olds Parent reported depression symptoms, punishing, distracting, and solicitous pain responses were not significantly associated with child or parent reported PI in the whole sample, 6–11-year-olds, or 12–17-year-olds
Klotsche 2014 [58] Article	61 C 61 P	x̄ = 11 (3–17) --	66 --	Po:67; O:21; E:5; S:2; Ps:3; U:2	• PI (P) – VAS CHAQ (9 timepoints: baseline, 1 mos, 2 mos, 3 mos, 4 mos, 5 mos, 6 mos, 9 mos, and 12 mos)	• HRQOL Total, Emotional Functioning, School Functioning, & Social Functioning (--) – PedsQL (9 timepoints)	• Univariate and Multivariate Reg controlling for disease activity, joints, stiffness, disability, & comorbidities – Lower well-being at baseline was significantly associated with greater PI at baseline • Latent Growth Curve Mixture Modelling – A rapid increase in well-being across the first 4 timepoints was significantly associated with lower PI at baseline • Linear Reg – Lower PI across timepoints significantly predicted better total well-being across time Lower PI across timepoints 1–7, but not 8 and 9 significantly predicted better emotional functioning across time Lower PI across timepoints 1–8, but not 9, significantly predicted better school and social functioning across time
Kovalchuk 2017 [59] Article	55 C 55 P	* (6–17) --	53 --	Po:53; O:47	• PI (P) – VAS CHAQ	• HRQOL Psychosocial (P) – CHQ	• Corr – Psychosocial well-being was not significantly associated with PI
Kovalchuk 2018 [60] Article	60 C 60 P 60 HCP	x̄ = 13 (5–17) -- --	48 100 --	Po:48; O:52	• PI (C & P) – VAS (current)	• HRQOL Behavior, Global Behavior, Self-Esteem, Family Cohesion, Family Activities, Mental Health, Time Impact, Emotional Impact, Emotional Role Limitations, Physical Role Limitations, & Psychosocial (P) – CHQ • Well-being (P, HCP, & C) – Global Assessment VAS	• Corr – Behavior, global behavior, self-esteem, family cohesion, mental health, and psychosocial summary scores were not significantly associated with parent or child reported PI Reduced engagement in family activities and greater impact on parents’ time and emotions were significantly associated with parent (but not child) reported PI More emotional and physical role limitations in parents, and lower parent, child, and healthcare provider global assessments of well-being were significantly associated with greater parent and child reported PI
Listing 2018 [62] Article	953 C 953 P	x̄ = 8 (–) --	67 --	Po:28; O:46; E:11; S:4; Ps:4; U:8	• PI (P) – NRS	• HRQOL (--) – PedsQL	• LR – Greater well-being at baseline was significantly associated with lower PI at baseline • Stepwise Reg – Greater PI at baseline significantly predicted lower well-being at 36 mos
Lomholt 2013^††^ [64] Article	41 C	x̄ = 14 (8–17)	71	Po:44; O:24; E:5; S:22; Ps:5	• PF (C) – FPS-R (2x/day for 2 weeks) pain/pain-free groups	• Coping Behavioral Distraction, Cognitive Distraction, Catastrophizing, & Positive Self-Statements (C) – PCQ • Pain Beliefs of Control, Disability, & Harm (C) – SOPA	• Mann Whitney U-Test – Behavioral distraction, cognitive distraction, the use of positive self-statements, and beliefs of control did not significantly differ between the pain and pain-free groups Greater catastrophizing, beliefs of harm, and beliefs of disability were significantly higher amongst the pain group compared to the pain-free group
Luca 2017 [65] Article	17 C 17 P	* (4–7) --	* --	*	• PI (C) –SUPERKIDZ (current and past week)	• HRQOL (C & P) – PedsQL Arthritis	• Corr – Child reported and parent reported well-being were not significantly associated with current and past week PI, respectively
Mahler 2017 [66] Abstract	51 C 51 P	Mdn = 13 (6–16) --	76 --	Po:27; O:37; E:4; S:10; Ps:11; U:11	• PI (--) – VAS JAMAR (past week)	• Well-being (--) – WHO-5	• Corr – Child well-being was not significantly associated with PI
Margetić 2005 [67] Article	36 C	x̄ = 13 (8–16)	61	--	• PI (C) – VAS (current)	• Anxiety and Depression symptoms (C) – TSC-C	• Corr and Reg – Greater depression, but not anxiety symptoms, were significantly associated with greater PI
Oen 2009^§^ [69] Article	356 C 356 P	Mdn = 9 (0–17) --	66 --	Po:24; O:41; E:10; S:7; Ps:7; U:12	• PI (--) – VAS (baseline and 6 mos)	• Well-being (--) – Global Assessment VAS assessed at baseline and 6 mos later • HRQOL (--) – JAQQ assessed at baseline and 6 mos later	• Corr – Lower well-being (VAS & JAQQ) at baseline was significantly associated with greater PI at baseline • Univariate & Multivariate Reg controlling for number of joints affected, baseline JAQQ, and time since diagnosis – Greater PI at baseline predicted lower well-being (JAQQ) at 6 mos
Oen 2021^§^ [68] Article	561 C	Mdn = 10 (–) --	65 --	Po:23; O:41; E:15; S:5; Ps:6; U:10	• PI (C) – VAS (past week at diagnosis, 3–9 mos post, and during flares)	• HRQOL (C) – JAQQ psychosocial assessed at diagnosis, 3–9 mos post, and during flares • HRQOL (C) – QoML assessed at diagnosis, 3–9 mos post, and during flares	• Corr in SEM – Greater PI at diagnosis and 3–9 mos post diagnosis were significantly associated with lower well-being (JAQQ & QoML) at diagnosis and 3–9 mos post diagnosis, respectively Greater PI during flares was significantly associated with lower well-being (QoML but not JAQQ) during flares
Rashid 2018^‡‡^ [4] Article	851 C 851 P	Mdn = 8 (1–16) --	66 --	Po:29; O:48; E:5; S:6; Ps:8; U:3	• PI (--) – VAS PPQ (baseline, 6 mos, and annually up to 60 months) average and 3 pain trajectories: consistently low/ improved/consistently high	• Well-being (P) – Global Assessment VAS assessed at baseline, 6 mos, and annually • Depression symptoms (--) MFQ assessed at baseline, 6 mos, and annually	• Corr – Lower well-being and greater depression symptoms at baseline were significantly associated with greater PI at baseline and less change in PI over time Greater PI at baseline was significantly associated with less change in well-being within 6 mos Change in PI within 12 mos was not significantly associated with change in well-being over 12 mos • Multinomial LoR – Well-being was significantly lower in the consistently high and improved pain groups compared to the consistently low pain group, and well-being significantly increased over 6 mos in the improved pain group compared to the consistently low pain group. No other differences emerged Depression symptoms did not significantly differ across groups
Ross 1993 [70] Article	56 C 56 P	x̄ = 12 (7–17) --	73 --	Po:59; O:27; E:5; S:9	• PI (C) – VAS (3x/day for 28 days) mean	• Behavior (P) – CBCL • Depression symptoms (C) – CDI • Anxiety symptoms (C) – STAI-C • Distress (C) – CDI and STAI-C • P Maternal Distress (P) – Lanyon Psychological Screening Inventory • P Family Harmony (P) – FES	• Corr and HR controlling for range of motion, disease activity, joint activity, stiffness, number of joints affected, and other measured variables – Behavior was not significantly associated with PI Greater anxiety symptoms, child distress, and maternal distress were significantly associated with greater PI Greater depression symptoms were significantly associated with but not predictive of greater PI Greater family harmony was not associated with but predicted greater PI
Sällfors 2004 [71] Article	125 C	x̄ = 14 (10–17)	66	Po:46; O:53; S:1	• PI (C) – VAS (usual) • PI (C) – NRS PIS (4x/day for 1 week) • PF (C) – PIS (pain free days)	• Well-being (C) – VAS CHAQ • Absences from school (C) – CHAQ	• Corr and Stepwise Reg – Lower well-being was significantly associated with greater PI (VAS & PIS) and PF • Corr – More school absences were significantly associated with greater PI (VAS & PIS) and PF
Schanberg 2003^‡^ [2] Article	41 C	x̄ = 12 (8–17)	59	Po:59; E:7; S:27; Ps:7	• PI (C) – VAS PPQ 1x/day at baseline, follow up, and for 2 mos) • PF (C) – VAS PPQ (percentage of pain days)	• Depression symptoms (C) – CDI assessed at baseline • Anxiety symptoms, Social Concerns, Physiologic Anxiety, & Worry (C) – RCMAS assessed at baseline	• Corr – Depression symptoms were not significantly associated with PI Greater physiologic anxiety was significantly associated with greater PI and PF Greater total anxiety symptoms, social concerns, and worry were significantly associated with greater PF
Schanberg 2005^‡^ [72] Article	51 C	x̄ = 12 (8–17)	65	Po:63; E:8; S:24; Ps:6	• PI (C) – VAS PPQ (1x/day at baseline, follow up, and for 2 mos)	• Stress (C) – Daily Events Inventory assessed daily for 2 months • Mood (C) – FAS assessed daily for 2 mos • Social & School Activity Reduction (C) – RCMAS assessed daily for 2 mos	• Longitudinal Mixed Effects Models – Greater same day stress and lower same day mood were significantly associated with greater same day PI • LMM controlling for disability index, global assessment, sex, age, disease onset, stiffness, fatigue, mood, and stress – Social, but not school, activity reduction was significantly associated with greater PI
Selvaag 2003 [73] Article	116 C 116 P	x̄ = 9 (4–17) x̄ = 38 (--)	60 *	Po:35; O:51; E:3; S:4; Ps:6; U:1	• PI (P) – VAS	• HRQOL psychosocial (--) – CHQ	• Corr – Psychosocial well-being was not significantly associated with PI
Selvaag 2005 [74] Article	– 197 P	x̄ = 7 (1–16) --	61 --	Po:30; O:56; E:4; S:7; Ps:3	• PI (P) – VAS	• Well-being (P) – Global Assessment VAS	• Corr – Lower well-being was significantly associated with greater PI
Shelepina 2011 [75] Abstract	99 C	-- (14–17)	73	Po:49; O:16; E:15; S:19	• PI (P) – VAS	• Schooling location (C)– -- school/home	-- – Children who were taught at home without medical indication reported significantly higher PI compared to those taught at school
Stinson 2006–1^†^ [76] Thesis	76 C	x̄ = 13 (9–17)	78	Po:49; O:15; E:11; S:13; Ps:11; U:3	• PI (C) – E-ouch VAS (3x/day for 14 days) • PI (C) – NRS RPI (past week)	• Pain Unpleasantness & Pain Interference (C) – E-Ouch • Pain Unpleasantness, Pain Interference Total, Pain Interference Mood, Pain Interference Relationships, Pain Interference Schoolwork, & Pain Interference Sleep (C) – RPI • Coping via Approach, Distraction, & Emotion-Focused Avoidance (C) – PCQ • HRQOL Total & Psychosocial (C) – PedsQL • HRQOL Arthritis Total, Worry, & Communication (C) – PedsQL Rheumatology	• Corr – Greater pain unpleasantness (E-Ouch and RPI) was significantly associated with greater PI (E-Ouch and RPI) across both weeks Greater pain interference (E-Ouch and RPI total, mood, relationships, schoolwork, sleep) was significantly associated with greater PI (E-Ouch and RPI) Approach coping and distraction coping were not significantly associated with PI (E-Ouch) on either week Greater emotion focused avoidance coping was significantly associated with greater PI (E-Ouch) on week 2 but not week 1 Lower total well-being, lower psychosocial well-being, lower total arthritis well-being, and more worry were significantly associated with greater PI (E-Ouch) Communication was not significantly associated with PI (E-Ouch)
Stinson 2006–2 [76] Thesis	36 C	x̄ = 13 (8–17)	67	Po:28; O:39; E:11; S:11; Ps:6; U:6	• PI (C) – E-ouch VAS (3x/day for 31 days; at day 7 had joint injections) • PI (C) – NRS RPI (past week)	• Pain Unpleasantness & Pain Interference (C) – E-Ouch • Pain Unpleasantness & Pain Interference (C) – RPI • Coping via Approach, Distraction, & Emotion-Focused Avoidance (C) – PCQ • HRQOL Total & Psychosocial (C) – PedsQL • HRQOL Arthritis Total, Worry, & Communication (C) – PedsQL Rheumatology	• Corr – Greater pain unpleasantness (E-Ouch and RPI) and pain interference (E-Ouch and RPI) were significantly associated with greater PI (E-Ouch and RPI) Approach coping, avoidance coping, and emotion-focused avoidance coping were not significantly associated with PI (E-Ouch) Lower total well-being and total arthritis well-being were significantly associated with greater PI (E-Ouch) Psychosocial well-being, worry, and communication were not significantly associated with PI (E-Ouch)
Tarakci 2011 [79] Article	52 C	x̄ = 12 (8–17)	63	Po:52; O:29; E:8; S:4; Ps:6; U:2	• PI (C) – VAS CHAQ (past week)	• Depression symptoms (C) – CDI • Anxiety symptoms (C)—SCARED • Well-being (C) – CHAQ	• Corr – Depression and anxiety symptoms were not significantly associated with PI Lower well-being was significantly associated with greater PI
Tarkiainen 2019 [80] Article	-- 60 P	* (4–14) --	65 --	Po:85; E:13; Ps:2	• PI (--) – VAS (8 × over 1 year)	• HRQOL psychosocial (C) – CHQ assessed 8 times throughout 1 year	• Univariate LMM – Greater PI was significantly associated with less improvement psychosocial well-being over time
Thastum 1997 [81] Article	15 C 15 P	x̄ = 12 (9–15) --	73 --	Po:20; O:80	• PI (C) – VAS (current) • PS (C) – Tolerance/time hand submerged • PS (C) – Threshold/time moved to button	• Coping via Catastrophizing, Distraction, & Reinterpretation (C) – preliminary PCQ	• Reg – Greater catastrophizing was significantly associated with greater PI and lower pain threshold (PS), but not pain tolerance (PS) Distraction and reinterpretation were not significantly associated with PI or PS (tolerance or threshold)
Thastum 1998 [82] Article	40 C	* (8–17)	58	--	• PI (C) – VAS PPQ (current, average, worst) high (modest disease activity and pain)/low (disease activity but few pain complaints) • PS (C) – Tolerance/ time hand submerged	• Coping via Behavioral Distraction, Cognitive Distraction, Information Seeking, Seeking Social Support, Externalizing, Catastrophizing, & Positive Self-Statements (C) – PCQ	• Corr and T-test – Greater behavioral distraction was significantly associated with lower PI (average, current, worst) but not experimental PI or PS. Behavioral distraction was significantly higher in the high pain group Cognitive distraction, information seeking, and seeking social support were not significantly associated with PI nor did it differ between high and low pain groups Greater externalizing was significantly associated with lower PS (i.e., higher tolerance); however, was not significantly associated with average, current, or worst PI (current and experimental) and did not differ between high and low pain groups Greater catastrophizing was significantly associated with greater experimental PI; however, was not significantly associated with average, current, or worst PI and did not differ between high and low pain groups Fewer positive self-statements were significantly associated with greater PI (average, current, worst); however, were not significantly associated with experimental PI and PS, and did not differ between high and low pain groups
Thastum 2005^††^ [25] Article	56 C	x̄ = 11 (7–15)	80	Po:41; O:43; E:2; S:13; Ps:2	• PI (C) – FPS (2x/day for 3 weeks) Mean and high (pain ≥ 1.61 & disease activity < 3)/low (remaining sample)	• Coping via Behavioral Distraction, Positive Self-Statements, Seeking Social Support, Cognitive Distraction, Catastrophizing, Externalizing, & Total (C) – PCQ • Pain Beliefs of Control, Harm, Disability, Solicitude, Medical Cure, Emotion, Total, Cognitive (disability + control + medical cure + harm), & Emotional (medical cure + emotion + solicitude) (C) – SOPA	• Corr and T-Test – Behavioral distraction and seeking social support were not significantly associated with PI, and they did not differ between high and low pain groups Positive self-statements were not significantly correlated with PI; however, were significantly higher in the low pain group • Corr, HR controlling for age, sex, disease duration, disease severity, disability, and pain beliefs, and T-Test – Cognitive distraction and externalizing were not significantly associated with PI and they did not differ between high and low pain groups Greater catastrophizing was significantly associated with greater PI (Corr, not Hierarchical Reg), and was significantly higher in the high pain groups • Corr and T-Test – Lower control beliefs were significantly associated with greater PI and were significantly lower in the high pain group Greater harm and disability beliefs were significantly associated with greater PI and were significantly higher in the high pain group Emotion beliefs were not significantly associated with PI nor did they differ between high and low pain groups Lower medical cure beliefs and higher solicitude beliefs were significantly associated with greater PI; however, did not differ between the high and low pain groups • HR controlling for age, sex, disease duration, disease severity, disability and pain coping – Worse pain beliefs (including cognitive beliefs but not emotional beliefs) were significantly associated with greater PI
Thastum 2011^††^ [83] Article	47 C	* (7–15)	83	Po:40; O:45; S:13; Ps:2	• PI (C) – FPS (2x/day for 3 weeks at baseline and 24 mos) Average and high (pain ≥ 1.61 & disease activity < 3)/low (remainder)	• Pain Beliefs of Control, Medical Cure, Harm, Disability, & Cognitive (disability + control + medical cure + harm) (C) – SOPA	• Corr – Lower control beliefs at baseline and 24 mos were significantly associated with greater PI 24 mos later Medical cure beliefs at baseline and 24 months were not significantly associated with PI at 24 mos • Corr and T-test – Greater harm and disability beliefs at baseline and 24 mos were significantly associated with greater PI 24 mos later, and significantly higher in the high pain group at 24 mos • HR controlling for disability, disease activity (and with/without baseline PI) – Greater cognitive beliefs at baseline significantly predicted PI at 24 mos
Thompson 1987 [26] Article	23 C 23 P	x̄ = 10 (5–15) --	78 100	Po:48; O:22; S:26; U:4	• PI (C) – VAS PPQ (current, worst, and high/low)	• Number of elevated behavior and social competence subscales, Overall Adjustment, Externalizing, Internalizing & Social Competence (P) – CBCL • P Family Relationships, Achievement, Active-Recreational Orientation, Cohesion, Conflict, Control, Expressiveness, Independence, Intellectual-Cultural Orientation, Moral-Religious Emphasis, & Organization (P) – FES	• Welch’s V – Children with 0, 1, 2, or 3 elevated behavior or social competence subscales did not significantly differ in current and worst PI • Corr and Welch’s V – Overall Adjustment, externalizing, internalizing, social competence, family relationships, conflict, active-recreational orientation, control, moral-religious emphasis, and organization were not significantly associated with PI, nor did they significantly differ between high and low pain groups Lower family achievement orientation was significantly associated with greater current, but not worst, PI, and it did not significantly differ between high and low pain groups Lower family cohesion and expressiveness were significantly associated with greater worst, but not current, PI, and they did not significantly differ between high and low pain groups Lower family independence and intellectual-cultural orientation were significantly associated with greater current, but not worst, PI, and they did not significantly differ between high and low pain groups
Tupper 2012 [84] Thesis	11 C	* (8–17)	*	Po:45	• PI (C) – VAS PinGo (7x/day for 4 days) 4 categories: 0 = None, 1–30 = Mild, 31–69 = Moderate, 70–100 = Severe	• Emotional valence (C) – FAS	• GEE – There was a significantly greater probability of having no pain during times of high emotional valence (regardless of activation level)
Tupper 2013^†^ [3] Article	85 C	x̄ = 13 (8–17)	73	Po:42; O:22; E:9; S:14; Ps:7; U:5	• PI (C) – E-ouch VAS (3x/day for 7 days)	• HRQOL (C)—PedsQL	• LR controlling for disease activity, illness duration, age, and sex – Greater PI variability was significantly associated with lower well-being
Upadhyay 2021 [85] Article	16 C	x̄ = 13 (8–16)	69	Po:81; O:13; Ps:6	• PI (C) – NRS PROMIS average and low (0–3)/high (> 3)	• Anxiety symptoms, Cognitive symptoms, Depression symptoms, and stress symptoms (C) – PROMIS	• Corr and T-test – Anxiety symptoms, depression symptoms, and stress were not significantly associated with PI, nor did they significantly differ between high and low pain groups Lower cognitive function was significantly associated with greater PI, although it did not significantly differ between high and low pain groups
Vandvik 1990 [86] Article	57 C 57 P	-- (7–16) --	67 --	Po:32; O:32; U:37	• PI (C) – VAS	• Psychosocial functioning (P) – CGAS • Overall adjustment, Externalizing, & Internalizing (P) – CBCL	• Corr – Psychosocial functioning, overall adjustment, externalizing, and internalizing were not significantly associated with PI
Vuorimaa 2008^§§^ [89] Article	145 C	x̄ = 12 (8–15)	73	Po:50; O:40	• PF (C) – SPQ (past 3 months)	• Trait anxiety symptoms (C) – STAI-C • Depression symptoms (C) – CDI • Children were categorized into: 1) teenagers high in trait anxiety and depression; 2) children high in trait anxiety and low in depression; 3) children low in trait anxiety and depression; and 4) teenagers low in trait anxiety and depression	• Discriminant Analyses – Cluster 1 (teenagers high in anxiety and depression symptoms) experienced significantly greater PF compared to the other clusters
Vuorimaa 2009^§§^ [88] Article	142 C 142 P	x̄ = 12 (8–15) *	73 83	Po:50; O:50	• PI (P) – VAS (current)	• Trait anxiety symptoms (C) – STAI-C • Depression symptoms (C) – CDI • Children were categorized into: 1) teenagers high in trait anxiety and depression; 2) children high in trait anxiety and low in depression; 3) children low in trait anxiety and depression; and 4) teenagers low in trait anxiety and depression	• Discriminant Analyses – Cluster 1 (teenagers high in anxiety and depression) experienced significantly greater PI compared to the other clusters
Vuorimaa 2011^§§^ [87] Article	142 C 142 P	x̄ = 12 (8–15) *	73 83	Po:50; O:50	• PF (C) – SPQ (past 3 months)	• Depression symptoms (C) – CDI • Anxiety symptoms (C) STAI-C • Psychological, Somatic, & Social Self-Efficacy (C) – CASE • P Depressive Symptoms (P) – BDI and HADS • P Anxiety Symptoms (P) – HADS • P Psychological, Social, & Somatic Self-Efficacy (C) – PASE • P Parent Influence on Child Mood, Parent Perception of Child’s Coping, & Parent Perception of Child’s Well-being (P) – Author created	• Corr and MR – Greater child depression and anxiety symptoms, lower child social self-efficacy, lower parent social self-efficacy, lower parent somatic self-efficacy, lower parent perception of the child’s well-being, and lower parent perception of the child’s coping were significantly associated with greater PF Greater parent depression symptoms (not MR with HADS) were significantly associated with greater PF Child psychological self-efficacy, child somatic self-efficacy, parent anxiety symptoms, parent psychological self-efficacy, and parent influence on child’s mood were not significantly associated with PF
Yan 2020 [91] Article	148 C	x̄ = 14 (8–17)	77	Po:18; O:53; E:13; S:7; Ps:2; U:7	• PI (C) – NRS PROMIS (past week; multiple visits)	• Depression symptoms (C) – PROMIS assessed across multiple visits	• LMM – Increasing PI was significantly associated with an increase in depression symptoms

Underlined text represents significant results. See Table 1 for master list of questionnaires and abbreviations

*ANOVA* Analysis of Variance, *ANCOVA* Analysis of Covariance, *C* Child, *Corr* Correlation, *E* Enthesitis-Related Arthritis, *GEE* Generalized Estimating Equations, *HCP* Healthcare providers, *HR* Hierarchical Regression, *LiR* Linear Regression, *LMM* Linear Mixed Models, *LoR* Logistic Regression, *MLM* Multilevel Models, *MR* Multiple Regression, *O* Oligoarticular Arthritis, *P* Parents/Caregivers, *Po* Polyarticular Arthritis, *Ps* Psoriatic Arthritis, *PF* Pain frequency, *PI* Pain intensity, *PS* Pain sensitivity/lower tolerance, *Reg* Regression, *S* Systemic Arthritis, *SEM* Structural Equation Models, *U* Undifferentiated/Other Arthritis

^‡, ‡‡, †, ††, §, §§^ Studies with overlapping datasets

^*^ Data provided but not specific to sample used in this review

-- Not reported

### Aim 1: Psychosocial correlates

#### Primary appraisals

##### Child correlates

There were 5 studies reporting on 28 associations between primary appraisals and pain in children with JIA. Pain unpleasantness was positively associated with pain intensity in 5/5 associations (herein referred to as 5/5) [76]. Pain beliefs were significantly associated with pain intensity (14/20) [25, 83] and pain frequency (2/3) [64]. Specifically, beliefs that pain causes harm and disability were positively associated with pain (5/5 each). Beliefs that one lacks control over their pain were positively associated with pain intensity (3/3) but not frequency (0/1). Beliefs there is no cure and that others should help with their pain (i.e., solicitude) were partially associated with pain intensity (1/3 and 1/2, respectively); whereas beliefs that emotions affect pain were not (0/2).

Taken together, although primary appraisals have been studied infrequently, perceptions of pain unpleasantness and beliefs that pain causes harm, disability, and loss of control appear to be consistently related to worse pain experiences in youth with JIA.

#### Secondary appraisals – internal factors

##### Child correlates

There were 7 studies reporting on 22 associations between internal factors a child may consider in their secondary appraisal and JIA pain, 8 of which were significant. Self-efficacy was negatively associated with pain intensity in 3/3 associations. Barlow, Shaw, and Wright [44] developed a measure to assess self-efficacy in children with arthritis. Each of the subscales (activity, emotion, and symptom) demonstrated a significant negative correlation to pain intensity. Vuorimaa and colleagues [87] used the same measure (with a different factor structure [151]) in relation to pain frequency, wherein 2/6 associations were significant (i.e., social self-efficacy but not psychological or somatic self-efficacy). Four additional internal factors were explored in relation to JIA pain. Neither children’s perceptions of their physical appearance (0/3) [42] nor child- or parent-reported self-esteem (0/4) [42, 60] were associated with pain intensity. Stress was positively related to pain intensity in 2/4 associations [51, 72, 85]; however, it is worth noting that nonsignificant results were only observed in one study with a small sample size (*n* = 16). Interestingly, difficulties with cognitive functioning were negatively correlated with pain intensity in select analyses (1/2) [85].

##### Parent correlates

Four studies reported on 17 associations between parent cognitive factors and pain in children with JIA. Of those, 8/17 were significant. Parent self-efficacy was negatively associated with pain intensity in 4/10 associations [43, 45] and pain frequency in 4/6 associations [87]. Specifically, psychosocial and symptom self-efficacy were negatively related to child pain intensity in 3/5 and 1/5 associations, respectively [43, 45]. Somatic and social self-efficacy, but not psychological self-efficacy, were negatively related to child pain frequency in 2/2 associations each [87]. Parent self-esteem was not related to children’s JIA pain (0/1) [60].

Taken together, despite the small sample sizes used in many of these studies, various domains of parent and child self-efficacy and children’s perceptions of stress have shown important associations to children’s JIA pain experiences.

#### Secondary appraisals – external factors

##### Child correlates

Sixteen studies reported on the relationship between social factors (i.e., school and social functioning, parent responses to pain, family functioning) and pain in children with JIA, with 30/105 significant associations. School functioning was significantly associated with pain intensity in 13/19 associations [40, 42, 52, 58, 71, 72, 75] and pain frequency in 1/1 association [71]. Greater pain was associated with more school absences or reduced school activity (6/8) [40, 52, 71] and home-schooling compared to traditional schooling (1/1) [75]. Pain did not appear to be associated with children’s perceptions of their scholastic competence (0/3) [42]. Similarly, social functioning and pain were significantly related in 9/35 associations. More specifically, social functioning was significantly associated with pain intensity in 8/34 associations [26, 42, 54, 58, 72, 76] and frequency in 1/1 association [2]. Klotsche and colleagues [58] found decreases in pain over time predicted better school and social functioning across 7/8 timepoints within one year. Schanberg and colleagues [2] also found a positive correlation between social concerns and pain frequency, and that pain scores were associated with increased odds of foregoing social activity (2/2) [2, 72]. No other associations were significant between pain and components of social functioning including social support, competence, skills, self-control, acceptance, communication, assertion, cooperation, or empathy (0/25) [26, 42, 54, 76].

Five studies reported on relationships between parent specific resources and children’s pain intensity, all of which had a sample size of less than 60 parents. Parent influences on the child’s mood [87] and responses to the child’s pain [57] were not associated with pain frequency or intensity (0/11); however, the measures used were not validated in this population. Family factors were variably related to pain intensity [26, 60, 70]. In some analyses, independence (1/3), achievement orientation (1/3), intellectual-cultural orientation (1/3), activities (1/2), cohesion (1/5), and expressiveness (1/3) were negatively associated with pain intensity, whereas harmony (1/2) was a positive relationship. Other factors including conflict, control, relationships, moral-religious emphasis, active-recreational orientation, and organization demonstrated no relationships (0/18).

Taken together, JIA pain is consistently associated with lower school and social functioning, but less related to actual skills. Although parent and family factors demonstrated less of a relationship, the studies included used small sample sizes and adapted measures.

#### Coping

##### Child correlates

Pain coping strategies were frequently assessed, and significantly associated with pain intensity in 15/61 associations [25, 46, 51, 76, 81, 82], pain frequency in 3/6 associations [64, 87], and pain sensitivity in 2/21 associations [50, 81, 82]. Greater coping ability and efficacy were negatively associated with pain (3/3) [47, 87]. Distraction is often cited as an adaptive coping strategy; however, only behavioral distraction was negatively associated with pain (4/9) [25, 64, 82]. Neither broad measures of distraction (0/6) [76, 81] nor measures of cognitive distraction (0/9) [25, 64, 82] were associated with pain. The use of positive self-statements is also presumed to be an adaptive coping style and was negatively associated with pain intensity (but not frequency or sensitivity) in 4/9 associations [25, 64, 82]. Catastrophizing is often cited as a maladaptive coping strategy, which was positively associated with pain intensity, frequency, and sensitivity in 7/22 associations [25, 50, 51, 64, 81, 82]. The remaining coping strategies were minimally or not associated with pain: externalizing (1/9) [25, 82]; emotion focused avoidance (1/2) [76]; and seeking social support, information seeking, approach, and reinterpretation (0/19) [25, 76, 81, 82]. Many studies exploring pain coping had relatively small sample sizes, likely contributing to the heterogeneity in results.

Taken together, despite some variability, children’s coping strategies of catastrophizing, behavioral distraction, and positive self-statements tended to show an important relationship to JIA pain.

#### Outcomes

##### Child correlates

Forty-two studies reported on 183 associations between pain and outcomes such as pain interference, mental health, and well-being, 104 of which were significant. Although a comprehensive review of the physical/functional limitations imposed by JIA pain were beyond the scope of this review, three studies found that the interference that pain imposed on daily activities was positively associated with pain intensity in 13/13 associations [60, 76].

Broad measures of child mental health were not significantly associated with pain intensity (0/8 associations) [26, 41, 60, 86] or sensitivity (0/8) [50]. Externalizing symptoms (e.g., behavior) were also not associated with pain intensity (0/12) [26, 42, 60, 70, 86], a finding that was stable across measures, reporters (parent, child), sample sizes (i.e., 23–60), and analyses (e.g., correlations, regressions). Internalizing symptoms (e.g., distress, emotional functioning) were positively associated with pain intensity in 10/16 associations [26, 39, 51, 58, 70, 86, 88] and with pain frequency in 1/1 association [89]. Most of the nonsignificant relationships used a proxy report to measure internalizing symptoms. Anxiety symptoms were positively associated with pain in 11/23 associations. More specifically, anxiety symptoms were positively associated with pain intensity in 4/10 associations [2, 67, 70, 76, 79, 85], pain frequency in 5/5 associations [2, 87], and pain sensitivity in 2/8 associations [50]. Across these studies, nonsignificant relationships tended to be more prevalent in studies with smaller sample sizes (i.e., 6/10 associations where n ≤ 52). Depression symptoms were positively associated with pain in 21/44 associations. Specifically, depression symptoms were positively associated with pain intensity in 19/42 associations [2, 4, 47, 49, 53–55, 57, 67, 70, 79, 84, 85, 91] and pain frequency in 2/2 associations [87]. While most scales assessed various depression symptoms (e.g., Children’s Depression Inventory, Mood and Feelings Questionnaire), some studies explored individual symptoms. Negative affect [47, 84], but not hopelessness or sadness [54], was found to be positively associated with greater pain intensity. Using a daily diary methodology, Connelly and colleagues [49] explored the relationship between emotion regulation and pain intensity. Although lower pain intensity was not correlated with child- or parent-reported emotion regulation or the adaptive upregulation of positive emotions, findings suggested that children with lower pain intensity were better able to manage their negative emotions and had fewer mood fluctuations day-to-day (i.e., less variability in positive and negative affect). Two studies explored the impact of pain on depression symptoms longitudinally. Hanns [55] found that higher baseline pain intensity was associated with worse depression symptoms over 12 months; results that were in keeping with other studies [91]. Across these associations, nonsignificant results were common in studies published before the year 2000; however, these studies also tended to report on younger samples (e.g., childhood) and used parent reports of depression symptoms (i.e., 7/7).

Greater HRQOL was significantly associated with lower pain intensity (28/37) [38, 58–60, 62, 65, 68, 69, 73, 76, 79, 80, 86] and lower pain intensity variability (1/1) [3], and greater well-being was significantly associated with lower pain intensity (15/16) [4, 60, 66, 69, 71, 74, 79] and pain frequency (4/4) [71, 87]. These findings were consistent across measures (e.g., Childhood Health Assessment Questionnaire, Pediatric Quality of Life Inventory), timeframes (e.g., usual, past week), reporters (child, parent, HCP), and analyses (e.g., correlations, regressions). In addition to cross-sectional studies, Listing and colleagues [62] found that greater pain intensity at baseline was not only associated with lower HRQOL at baseline, but also 36 months later. Similar results were found by others [58, 69, 80]. Nonsignificant results were more prevalent in studies with small sample sizes (i.e., 3/5 studies where n ≤ 36) and those assessing psychosocial HRQOL especially with the Child Health Questionnaire (7/11 studies).

##### Parent correlates

Six studies reported on 22 associations between parent mental health outcomes and JIA pain. Mothers’ mental health was over-represented (samples ranged from 83 to 100% female). Across these studies, 9/22 associations were significant. Parent internalizing symptoms (e.g., distress) were positively related to child pain intensity in 2/3 associations [48, 70]. Parental symptoms of anxiety were not associated with child pain intensity or frequency (0/3) [45, 87]. Parental symptoms of depression were positively associated with pain frequency (3/4) [87], but not intensity (0/2) [39, 45]; however, the latter two studies had smaller sample sizes (*n* ≤ 51). Parent identified limitations that pain imposed on their daily activities were positively associated with their child’s pain in 4/10 associations [39, 48, 60]. More specifically, Bruns and colleagues [48] were unable to demonstrate a relationship between caregiver burden and child pain intensity; however, Kovalchuk and colleagues [60] found that time and emotional impact were positively correlated with parent- (but not child-) reported pain intensity. Furthermore, Anthony and colleagues [39] found that although parent-reported hassles (i.e., perceptions of daily events like the weather and their workload as negative) were not significantly associated with child pain intensity, the frequency of parent-reported uplifts (i.e., parents identifying daily events as positive) was interestingly associated with greater child-reported pain.

Taken together, internalizing symptoms in children (anxiety, depression, and interference) and parents (depression, impacts on time and emotions, and more frequent uplifts) tend to demonstrate reliable associations to greater pain in children in studies with sufficient sample sizes using validated self-report measures, whereas greater HRQOL/well-being appears to be robustly related to lower JIA pain in children with JIA.

### Aim 2: Prognostic factors

#### Primary appraisals

##### Child factors

The relationship between pain beliefs and pain were assessed prognostically in one study [83], wherein 4/5 associations were significant. Following up on their earlier work, Thastum and Herlin [83] explored the impact of pain beliefs on pain intensity two years later. They found that baseline beliefs of harm, disability, and lack of control (but not that there is no medical cure) were positively correlated with later pain intensity, and that cognitive beliefs (i.e., the sum of the above beliefs) predicted greater pain intensity two years later. Taken together, pain beliefs are an important prognostic factor for later JIA pain experiences.

#### Outcomes

##### Child factors

Prognostically, the relationship between depression symptoms and pain intensity were explored in four studies [4, 49, 55, 56]. Of those, depression symptoms significantly predicted pain intensity in 7/14 associations. Connelly and colleagues [49] used a 28-day daily diary study to explore whether emotion regulation predicted pain intensity. Through linear mixed models, they found similar results longitudinally as were reported cross-sectionally. Namely, greater variability in positive and negative emotions predicted greater pain intensity over time, and the adaptive upregulation of positive emotions following a drop in emotions predicted lower pain intensity over time. Two studies using the same database [4, 55] found that more depression symptoms at baseline predicted greater pain intensity and less improvement in pain over at least one year. Rashid and colleagues [4] went on to conduct a group-based trajectory analysis, however no differences in depression symptoms across pain groups were observed. Finally, Hoff and colleagues [56] assessed depression symptoms and pain intensity dyadically over 12 months. Although child-reported baseline depression symptoms did not predict later parent-reported pain intensity, it predicted later child-reported pain intensity when pain was low at baseline.

The relationship between well-being and pain was also explored by Rashid and colleagues [4], wherein 4/8 associations were significant. Worse baseline well-being was significantly correlated with less change in pain intensity over 12 months; however, change in well-being was not correlated with change in pain intensity. Moreover, in their group-based analyses, the “consistently high” and “improved pain” groups had significantly worse baseline well-being than the “consistently low” pain group, and improvements in well-being at six months were more likely in the “improved pain” group compared to the “consistently low” pain group.

In sum, the predictive value of depression symptoms on later pain experiences appeared to be contingent on the specific symptoms assessed and the reporter of these variables. Nevertheless, greater depression symptoms and lower well-being were predictive of worse pain intensity over time, but both relationships are likely more complex.

## Discussion

Pain is a common experience that affects children with JIA in many ways. Across 61 studies, 516 unique associations between pain and psychosocial factors were identified. Most studies explored these associations cross-sectionally, with 51 associations explored longitudinally. The studies were of moderate quality, with the identification of confounds, and validity of outcome (i.e., pain) measures as the biggest areas for improvement. All studies were nevertheless included. Various factors were explored in relation to JIA pain, speaking to the complex relationships that exist; however, the emphasis was predominantly on child outcomes (e.g., mental health, well-being) and less on primary and secondary appraisals within the child and caregiver. Within and between studies, only a few variables were always related to JIA pain (unpleasantness and interference; beliefs of harm, disability, and control). The heterogeneity of most results is likely attributable to the moderate study quality, variability in measures and reporters, and small sample sizes; publication year did not appear to impact results substantially. Various factors are nevertheless important to consider as the associations were generally significant and trending in the same direction.

With regards to children’s primary appraisals, two constructs were looked at in relation to JIA pain – perceptions of pain unpleasantness and pain beliefs. These perceptions and beliefs are assumptions of reality through which events such as arthritis pain can be interpreted, and are thereby presumed to affect coping efforts and the pain experience [152]. For example, a child who believes their JIA pain is purely physical in nature may feel a lack of control over their pain, thus increasing the attention given to their pain experience. While only a few studies explored these associations, results consistently demonstrated that perceptions of unpleasantness and beliefs that pain signifies harm, causes functional disability, and is unable to be controlled were significantly associated with worse JIA pain cross-sectionally and longitudinally. Less consistently, beliefs that there is no cure, that emotions impact pain, and that others should respond solicitously tended to be associated with greater pain. Pain beliefs appear to be a promising area for future research, especially in conjunction with pain neuroscience as an intervention to target unhelpful beliefs.

A few constructs were explored pertaining to the child’s and parent’s assessments of their internal and external resources available to manage JIA pain (i.e., secondary appraisals). While some internal resources (self-esteem, cognitive functioning, stress, perceptions of physical appearance) were minimally explored, one was explored in greater depth. Self-efficacy is one’s expectations of success in performing the behaviors required to meet a specific outcome [153], which has theoretical implications for the actions one takes, the amount of effort exerted, and the nature of one’s thoughts and emotions [44]. It is thought to be a key mechanism of change in fostering resilience [154]. Although a relatively nascent construct in pediatric pain, within broader pain populations it has also been associated with lower pain severity [155]. Two teams explored self-efficacy in this population using different subscales and pain outcomes. Across these studies, both child and parent self-efficacy (albeit in different domains) were generally related to better pain experiences. Thus, self-efficacy is a vital construct for further exploration.

Various factors pertaining to external resources were also explored. While JIA pain was not associated with impaired social skills, it was generally associated with worse school (e.g., attendance, paying attention in class, keeping up with schoolwork) and social (e.g., getting along with others, having friends) functioning. These findings parallel the pain literature [156, 157] and can be understood through the interpersonal fear avoidance model of pain [158]. The child’s internal pain experience is theorized to lead to negative cognitions, which can contribute to avoidant behaviors (e.g., avoiding school or friends). This can limit the child’s social support which, upon future secondary appraisals, can further aggravate the child’s pain. Longitudinal designs are required to fully understand these pathways. This model also highlights how parents contribute to children’s pain experiences. Parent pain responses (e.g., responding protectively, reinforcing activity restriction, distracting) were not significantly related to JIA pain in this review, which is in line with a recent meta-analysis demonstrating that they are more closely related to functional disability [159]. Family variables (e.g., harmony, cohesion) have also been postulated to affect pain intensity in JIA; however, in this review, as in the broader literature [160], these relationships were unreliable. Pain was inconsistently associated with greater harmony and less achievement, achievement orientation, expressiveness, activities, cohesion, and intellectual-cultural orientation. It is possible that JIA pain may cause a unique dynamic within the family, wherein the family engages in fewer activities, is less cohesive, and is more co-dependent. Greater family harmony was an interesting finding, which was theorized to be because an overly harmonious and responsive environment may reinforce pain behaviors [70]. These results must be interpreted with caution given the small sample sizes of the studies exploring them. More research with larger samples, new pain-specific family measures, and longitudinal studies showing how family functioning varies with pain flares is warranted.

Coping, or the intentional use of thoughts and behaviors to manage stressful experiences [161], was also explored in relation to JIA pain. Certain coping strategies are posited to be adaptive and have the potential to improve the child’s well-being and pain experience (e.g., seeking information and social support, problem solving, positive self-statements, distraction). Other strategies are viewed as maladaptive and are thought to be associated with worse well-being and pain (e.g., emotion-focused avoidance, catastrophizing, externalizing; [162]). With that said, there is significant variability in the pediatric pain literature regarding coping theories, measures, and responses [163], which was also observed in this review. While the associations identified in this review trended in the expected directions, results were neither straightforward nor unanimous. Specifically, only positive self-statements and behavioral distraction were generally associated with reduced pain, and only catastrophizing tended to be associated with greater pain. Strategies such as seeking social support and information, externalizing, emotion-focused avoidance, and approach were not significant in either direction. These results are likely a function of the broader variability in the literature [163] as well as the small sample sizes of the included studies. Moreover, no studies investigated these findings longitudinally or explored parent coping. As such, there is a clear need for more theoretically-driven research understanding the role of child and parent coping in JIA pain cross-sectionally and longitudinally.

Outcomes in relation to JIA pain (e.g., mental health, well-being) were explored most frequently and are presumed to be a result of the primary and secondary appraisals and coping efforts and can subsequently influence future appraisals. One of the most consistent findings of this review was the negative relationship between pain and measures of HRQOL and well-being. Results were demonstrated cross-sectionally and longitudinally in both directions (i.e., pain predicting lower well-being and the reverse). In considering the multidimensional nature of pain, HRQOL comprises the evaluative component, or the way in which pain affects one’s broader well-being such as their functioning [164, 165]. Thus, the consistent and bidirectional relationships identified in this review are well grounded in the literature. Although nonsignificant results were observed, they were more prevalent in studies with smaller sample sizes and those using the Child Health Questionnaire (a measure reported to be confusing due to the varying response options and recall periods across items [166]). Although broad measures of child mental health and externalizing symptoms were not related to JIA pain, significant associations were often observed with measures assessing internalizing symptoms, and more specifically symptoms of interference, anxiety, and depression. Nonsignificant results tended to occur in younger samples, when proxy reports of internalizing symptoms or pain were used, and in studies with smaller sample sizes. As pain and internalizing symptoms are internal experiences, proxy reporters may not fully understand the child’s experiences with either, leading to null results. Nevertheless, these findings parallel what has been seen in the broader pediatric pain literature [167]. With regards to the relationships between pain and depression symptoms, interestingly results were retained in longitudinal designs, with some studies finding that pain predicted later depression symptoms, and other studies demonstrating the reverse. Current frameworks suggest that rather than one causing the other, there is a shared vulnerability wherein pain and internalizing symptoms may develop and maintain one another (see Jastrowski Mano [168], Soltani [169], and Vinall [170] for reviews).

The role of parent mental health is also salient in these frameworks. In this review, a small number of studies cross-sectionally explored the relationship between parent (largely maternal) mental health and JIA pain. Although anxiety symptoms were not related to pain, few studies examined this. Broader measures of internalizing and depression symptoms demonstrated a relationship to greater JIA pain in some but not all associations, as did scales assessing the impacts pain has on parents’ time and emotions. This is consistent with the small to null effects found in a recent meta-analysis on the role of parent factors in pediatric pain [171]. As suggested by the abovementioned frameworks, it is likely that the relationship does exist, however is more complex than correlations may suggest. According to social learning theory, a parent observing their child in pain may experience internalizing symptoms which through modelling and specific responses may contribute to the child’s own internalizing symptoms and draw greater attention to their pain experience. More research is needed to further test these frameworks, particularly as it relates to paternal mental health. Another interesting finding emerged, wherein more parent-reported uplifts, or positive events in the day, was associated with greater pain [39]. It was posited that increased pain led to parents being more attentive to positive daily experiences or that parents were more attentive to their child’s pain when there were more positive events in the day; however, future research is warranted to test these theories.

In sum, numerous psychosocial correlates have been identified in relation to JIA pain, all of which have important implications in the child’s future appraisals of JIA pain and are key targets for pain assessment and intervention. This study had strengths in its inclusion of multiple dimensions of the pain experience, a broad array of psychosocial factors, multiple reporters, and unlimited inclusion dates and quantitative designs. There are also limitations. The search was restricted to children 0–17 years of age; some studies were excluded because they included youth 18 years and older, thus limiting the scope of this review. Similarly, only studies that included “pain” or some variation of the term in their abstracts were included. It is possible that some studies were missed as they did not mention pain or used a different dimension of pain all together (e.g., impact, number of painful joints). Finally, given the heterogeneity of the associations and samples included, the focus was on significance and directionality. Future research may benefit from using effect sizes and meta-analytic techniques to further explore these relationships [167], though at present methodology and measurement is so diverse across studies that this may be premature.

The results of this review identify important research directions. Most studies assessed correlational relationships between psychosocial factors and JIA pain. To advance our understanding of factors predictive of JIA pain, there is a need for high quality longitudinal designs. With regards to methodological considerations, participants were largely females with polyarticular or oligoarticular JIA. Future research should seek to explore the pain experience in other populations such as males, other JIA subtypes, and diverse ethnic backgrounds. Furthermore, over 20 studies did not clearly cite or describe their pain measure, 15 relied on a proxy report of pain, and seven did not clarify who the reporter was. While some of these studies may have predated best practice in pediatric pain research, it is recommended that future studies obtain self-reports of pain in children ages 5–6 years old and older [172] and behavioral observations for younger or nonverbal children [173]. Assessment of pain in younger or nonverbal children nevertheless remains an important area where further research is required, especially in the context of JIA. These results similarly highlight the inconsistency in measures used to assess psychosocial factors, suggesting the need for greater consensus and psychometric support across measures in this population. Moreover, it is well known that these relationships are more complex than can be expressed through correlations or main effects. An important next step will be to use larger samples and/or open databases that allow for complex analyses that will offer insight into how biopsychosocial factors interact to affect JIA pain (e.g., functioning, rheumatoid factor, cyclic citrullinated peptide antibodies, the child’s growth and development, bone and mineral metabolism) [167], and how the relationship between psychosocial factors and pain may differ based on subgroups of individuals (e.g., the 10–15% of children with JIA who experience more chronic JIA [5, 174]). Finally, this review has highlighted a restricted set of psychosocial correlates, despite a call nearly 2 decades ago to explore the role of parent/family factors in relation to pain [175], and more recent calls to take a strengths-based approach [176]. As such, in addition to more rigorously assessing the identified associations, there are many factors that were not identified in this review and as such have yet to be explored in relation to JIA pain (e.g., parent factors, temperament/personality dimensions, resilience).

These findings have important clinical implications. Of primary importance is that pain should be assessed comprehensively and regularly in clinics. Stinson and Prescott have outlined several brief and validated pain assessment measures to use with youth diagnosed with JIA [165]. The psychosocial factors identified play an important role in the child’s pain experience, regardless of whether they cause, are caused by, or are only tangentially related to JIA pain. In line with the interdisciplinary approach to pain management, while pharmacological and physical strategies may be appropriate, psychosocial supports may also be warranted given these results. With regards to psychological interventions, there is preliminary support for their efficacy in reducing pain (and improving other outcomes) in children with JIA [27, 28]. The findings of this review can help refine and design new interventions tailored to address factors associated with worse pain and promote factors associated with reduced pain.

## Conclusions

JIA pain is a complex and pervasive issue. This study has identified psychosocial factors that tend to be associated with or predictive of JIA pain, including child pain beliefs, internal and external resources (e.g., self-efficacy, social factors, intervention participation), and outcomes such as internalizing symptoms and well-being. Results however should be interpreted with caution given the heterogeneity of findings. These results can help guide the clinical care of children with JIA and can better inform interventions. Moreover, this study has identified several directions for future research, including the use of validated pain measures and larger samples to explore the interactions amongst variables.

## Supplementary Information

**Additional file 1.** Search Strategy, Search terms used in specific databases.

**Additional file 2.** Data Extraction Template, Template of information extracted from included articles.

**Additional file 3:** **3.1.** Quasi-Experimental Studies, Results and Discussion of Quasi-Experimental Studies. **3.2.** Critical Appraisal Results for Quasi-Experimental Studies, Critical Appraisal Results Table for Quasi-Experimental Studies. **3.3.** Quasi-Experimental Study Characteristics and Results, Study Characteristics and Results Table for Quasi-Experimental Studies.

**Additional file 4.** Summary of Results, Summary of results in tabular form.

## Data Availability

The search string used to identify relevant studies in the current review is available in the supplementary materials. The search string has also been saved at searchrxiv.org.

## Abbreviations

CINAHL: Cumulative Index to Nursing and Allied Health Literature
HCP: Healthcare providers
JBI: Joanna Briggs Institute
JIA: Juvenile Idiopathic Arthritis
PRISMA: Preferred Reporting Items for Systematic Reviews and Meta-analysis
PROSPERO: International Prospective Register of Systematic Reviews
