# Supplementary material for: A systematic review of the psychosocial factors associated with pain in children with juvenile idiopathic arthritis
Source: Pediatr Rheumatol Online J. 2023 Jun 16;21:57. doi: 10.1186/s12969-023-00828-5 (PMC10273767; doi:10.1186/s12969-023-00828-5)
Supplement: Supplementary file 1 — Additional file 1. Search Strategy, Search terms used in specific databases. [file 12969_2023_828_MOESM1_ESM.docx]

Additional File 1: Search Strategy

| **Juvenile Idiopathic Arthritis terms** | **Pain terms [35]** | **Child terms [36]** |
| --- | --- | --- |
| Juvenile idiopathic arthritis  Juvenile arthritis  Juvenile chronic arthritis  Juvenile rheumatoid arthritis  Juvenile rhematic disease  Inflammatory arthropathy  Oligoarticular arthritis  Pauciarticular arthrit*  Polyarticular arthrit*  Systemic arthrit*  Enthesitis arthrit*  Psoriatic arthrit*  Undifferentiated arthrit* | Pain*  Hurt*  Discomfort*  Chronic pain  Acute pain  Procedural pain  Needle  Injection*  Syringe*  Pain perception  Nociception  Pain threshold  Hyperalgesi*  Hypoalgesi*  Enthesalgi*  Central sensitivity  Somatosensory profile Experimental pain Cold pressor Quantitative sensory test Water load Heat pain Thermal pain Pressure pain Exercise task  Pain management  Pain measurement | Infan*  Perinat*  Antepartum  Ante-partum  Postnatal*  Post-natal*  Baby*  Babies  Neonat*  Neo-nat*  Newborn*  New-born*  ﻿Child*  Kid  Kids  Toddler*  Girl*  Girls  Girlhood  Boy*  Boys  Boyhood  Preschool*  Pre-school*  Kindergarten*  School*  School child  Juvenil*  Minors*  P?ediatric?  Pe?diatric?  Pediatric*  Prepubescen*  Pre-pubescen*  Pubescen*  Primary school  Teen*  Youth*  Adolescen*  Young adult*  Young person*  Young individual*  Young people*  Young population*  Student*  Highschool*  High-school*  High school  Secondary school |
| **OVID Medline search format:**  Arthritis, juvenile/ OR Stills disease/ OR Spondyloarthropathies/ OR Spondylitis/ OR Spondylitis, Ankylosing/ OR Arthritis, Psoriatic/ OR ("Juvenile idiopathic arthrit*" OR "JIA" OR "Juvenile arthrit*" OR "JA" OR "Juvenile chronic arthrit*" OR "JCA" OR "Juvenile rheumatoid arthrit*" OR "JRA" OR "Juvenile rheumatic disease*" OR "Inflammatory arthropathy" OR "JRD" OR (Oligo* ADJ5 arthrit*) OR "Oligoarthrit*" OR "oJIA" OR "OligoJIA" OR (Pauci* ADJ5 arthrit*) OR (Poly* ADJ5 arthrit*) OR (Systemic* ADJ5 arthrit*) OR (Systemic-onset ADJ5 arthrit*) OR "S-JIA" OR "SJIA" OR "SO-JIA" OR "SOJIA" OR "Still? disease" OR "Still? syndrome" OR (Enthesit* ADJ5 arthrit*) OR (Enthesitis-related ADJ5 arthrit*) OR "Spondyloarthr?path*" OR "JuSpA" OR "JSpA" OR "Ankylosing spondylitis" OR "JAS" OR (Psoria* ADJ5 arthrit*) OR (Undifferentiated ADJ5 arthrit*)).ti,ab,kw,kf. | **OVID Medline search format:**  Pain/ OR Chronic Pain/ OR Pain, Intractable/ OR Acute Pain/ OR Pain, Procedural/ OR Injections/ OR Injections, Intramuscular/ OR Injections, Intra-articular/ OR Injections, Subcutaneous/ OR Injections, Intravenous/ OR Syringes/ OR Perception, Pain/ OR Nociception/ OR Pain Threshold/ OR Management, Pain/ OR Analgesia/ OR Analgesics/ OR Measurement, Pain/ OR ("Pain*" OR "Hurt*" OR "Discomfort*" OR "Chronic pain" OR "Acute pain" OR "Procedural pain" OR "Needle*" OR "Injection*" OR "Syringe*" OR "Experimental pain" OR "Cold pressor" OR "Quantitative sensory test*" OR "Water load" OR "Heat pain" OR "Thermal pain" OR "Pressure pain" OR "Exercise task" OR "Nocicepti*" OR "Pain* threshold*" OR "Hyperalgesi*" OR "Hypoalgesi*" OR "Enthesalgi*" OR "Central sensitivity" OR "Somatosensory profile*" OR "Pain* management*" OR "Analgesi*" OR "Pain* measurement*").ti,ab,kw,kf. | **OVID Medline search format:**  Exp Infant/ OR Exp Infant, Newborn/ OR Exp Behavior, Infant/ OR Exp Health, Infant/ OR Exp Child/ OR Exp Child, Preschool/ OR Exp Behavior, child/ OR Exp Health, Child/ OR Exp Pediatrics/ OR Exp Adolescent/ OR Exp Behavior, adolescent/ OR Exp Health, adolescent/ OR Exp Young Adult/ OR ("Infan*" OR "Perinat*" OR "Antepartum" OR "Ante-partum" OR "Postnatal*" OR "Post-natal*" OR "Baby*" OR "Babies" OR "Neonat*" OR "Neo-nat*" OR "Newborn*" OR "New-born*" OR "Child*" OR "Kid" OR "Kids" OR "Toddler*" OR "Girl*" OR "Girls" OR "Girlhood" OR "Boy" OR "Boys" OR "Boyhood" OR "Preschool*" OR "Pre-school*" OR "Kindergarten*" OR "School*" OR "Juvenil*" OR "Minors*" OR "P?ediatric?" OR "Pediatric*" OR "Prepubescen*" OR "Pre-pubescen*" OR "Pubescen*" OR (Primary ADJ2 school) OR (Primary ADJ2 education) OR "Teen*" OR "Youth*" OR "Adolescen*" OR (Young ADJ2 adult*) OR (Young ADJ2 person*) OR (Young ADJ2 individual*) OR (Young ADJ2 people*) OR (Young ADJ2 population*) OR "Student*" OR "Highschool*" OR "High-school*" OR (High ADJ2 school*) OR (Secondary ADJ2 school*)).ti,ab,kw,kf. |
| **CINAHL search format:**  ((MH "Arthritis, juvenile rheumatoid") OR (MH "Spondyloarthropathies") OR (MH "Spondlyarthritis") OR (MH "Spondylitis, ankylosing") OR (MH "Arthritis, Psoriatic") OR (TI ("Juvenile idiopathic arthrit*" OR "JIA" OR  "Juvenile arthrit*" OR "JA" OR "Juvenile chronic arthrit*" OR "JCA" OR "Juvenile rheumatoid arthrit*" OR "JRA" OR "Juvenile rheumatic disease*" OR "Inflammatory arthropathy" OR "JRD" OR (Oligo* N5 arthrit*) OR "Oligoarthrit*" OR "oJIA" OR "OligoJIA" OR (Pauci* N5 arthrit*) OR (Poly* N5 arthrit*) OR (Systemic* N5 arthrit*) OR (Systemic-onset N5 arthrit*) OR "S-JIA" OR "SJIA" OR "SO-JIA" OR "SOJIA" OR "Still# disease" OR "Still# syndrome" OR (Enthesit* N5 arthrit*) OR (Enthesitis-related N5 arthrit*) OR "Spondyloarthr#path*" OR "JuSpA" OR "JSpA" OR "Ankylosing spondylitis" OR "JAS" OR (Psoria* N5 arthrit*) OR (Undifferentiated N5 arthrit*))) OR (AB ("Juvenile idiopathic arthrit*" OR "JIA" OR  "Juvenile arthrit*" OR "JA" OR "Juvenile chronic arthrit*" OR "JCA" OR "Juvenile rheumatoid arthrit*" OR "JRA" OR "Juvenile rheumatic disease*" OR "Inflammatory arthropathy" OR "JRD" OR (Oligo* N5 arthrit*) OR "Oligoarthrit*" OR "oJIA" OR "OligoJIA" OR (Pauci* N5 arthrit*) OR (Poly* N5 arthrit*) OR (Systemic* N5 arthrit*) OR (Systemic-onset N5 arthrit*) OR "S-JIA" OR "SJIA" OR "SO-JIA" OR "SOJIA" OR "Still# disease" OR "Still# syndrome" OR (Enthesit* N5 arthrit*) OR (Enthesitis-related N5 arthrit*) OR "Spondyloarthr#path*" OR "JuSpA" OR "JSpA" OR "Ankylosing spondylitis" OR "JAS" OR (Psoria* N5 arthrit*) OR (Undifferentiated N5 arthrit*)))) | **CINAHL search format:**  ((MH "Pain") OR (MH "Chronic pain") OR (MH "Acute pain") OR (MH "Pain, procedural") OR (MH "Treatment related pain") OR (MH "Injections") OR MH (“Injections, intramuscular") OR (MH "Injections, intra-articular") OR (MH "Injections, subcutaneous") OR (MH "Injections, intravenous") OR (MH "Syringes") OR (MH "Nociceptive Pain") OR (MH "Pain threshold") OR (MH "Pain management") OR (MH "Analgesia") OR (MH "Analgesics") OR (MH "Pain measurement") OR (TI ("Pain*" OR "Hurt*" OR "Discomfort*" OR "Chronic pain" OR "Acute pain" OR "Procedural pain" OR "Needle*" OR "Injection*" OR "Syringe*" OR "Experimental pain" OR "Cold pressor" OR "Quantitative sensory test*" OR "Water load" OR "Heat pain" OR "Thermal pain" OR "Pressure pain" OR "Exercise task" OR "Nocicepti*" OR "Pain* threshold*" OR "Hyperalgesi"*" OR "Hypoalgesi"*" OR "Enthesalgi*" OR "Central sensitivity" OR "Somatosensory profile*" OR "Pain* management*" OR "Analgesi*" OR "Pain* measurement*")) OR (AB ("Pain*" OR "Hurt*" OR "Discomfort*" OR "Chronic pain" OR "Acute pain" OR "Procedural pain" OR "Needle*" OR "Injection*" OR "Syringe*" OR "Experimental pain" OR "Cold pressor" OR "Quantitative sensory test*" OR "Water load" OR "Heat pain" OR "Thermal pain" OR "Pressure pain" OR "Exercise task" OR "Nocicepti*" OR "Pain* threshold*" OR "Hyperalgesi*" OR "Hypoalgesi*" OR "Enthesalgi*" OR "Central sensitivity" OR "Somatosensory profile*" OR "Pain* management*" OR "Analgesi*" OR "Pain* measurement*”))) | **CINAHL search format:**  ((MH "Infant+") OR (MH "Infant, Newborn+") OR (MH "Infant Behavior") OR (MH "Child+") OR (MH "Child, Preschool") OR (MH "Child Behavior+") OR (MH "Child Health") OR (MH "Pediatrics+") OR (MH "Adolescence+") OR (MH "Adolescent behavior") OR (MH "Adolescent health") OR (MH "Young adult") OR (TI ("Infan*" OR "Perinat*" OR "Antepartum" OR "Ante-partum" OR "Postnatal*" OR "Post-natal*" OR "Baby*" OR "Babies" OR "Neonat*" OR "Neo-nat*" OR "Newborn*" OR "New-born*" OR "Child*" OR "Kid" OR "Kids" OR "Toddler*" OR "Girl*" OR "Girls" OR "Girlhood" OR "Boy*" OR "Boys" OR "Boyhood" OR "Preschool*" OR "Pre-school*" OR "Kindergarten*" OR "School*" OR "Juvenil*" OR "Minors*" OR "P?ediatric?" OR "Pediatric*" OR "Prepubescen*" OR "Pre-pubescen*" OR "Pubescen*" OR (Primary N2 school) OR (Primary N2 education) OR "Teen*" OR "Youth*" OR "Adolescen*" OR (Young N2 adult*) OR (Young N2 person*) OR (Young N2 individual*) OR (Young N2 people*) OR (Young N2 population*) OR "Student*" OR "Highschool*" OR "High-school*" OR (High N2 school*) OR (Secondary N2 school*)) OR (AB ("Infan*" OR "Perinat*" OR "Antepartum" OR "Ante-partum" OR "Postnatal*" OR "Post-natal*" OR "Baby*" OR "Babies" OR "Neonat*" OR "Neo-nat*" OR "Newborn*" OR "New-born*" OR "Child*" OR "Kid" OR "Kids" OR "Toddler*" OR "Girl*" OR "Girls" OR "Girlhood" OR "Boy*" OR "Boys" OR "Boyhood" OR "Preschool*" OR "Pre-school*" OR "Kindergarten*" OR "School*" OR "Juvenil*" OR "Minors*" OR "P?ediatric?" OR "Pediatric*" OR "Prepubescen*" OR "Pre-pubescen*" OR "Pubescen*" OR (Primary N2 school) OR (Primary N2 education) OR "Teen*" OR "Youth*" OR "Adolescen*" OR (Young N2 adult*) OR (Young N2 person*) OR (Young N2 individual*) OR (Young N2 people*) OR (Young N2 population*) OR "Student*" OR "Highschool*" OR "High-school*" OR (High N2 school*) OR (Secondary N2 school*))) |
| **PsycINFO search format:**  (TI ("Juvenile idiopathic arthrit*" OR "JIA" OR "Juvenile arthrit*" OR "JA" OR "Juvenile chronic arthrit*" OR "JCA" OR "Juvenile rheumatoid arthrit*" OR "JRA" OR "Juvenile rheumatic disease*" OR "Inflammatory arthropathy" OR "JRD" OR (Oligo* N5 arthrit*) OR "Oligoarthrit*" OR "oJIA" OR "OligoJIA" OR (Pauci* N5 arthrit*) OR (Poly* N5 arthrit*) OR (Systemic* N5 arthrit*) OR (Systemic-onset N5 arthrit*) OR "S-JIA" OR "SJIA" OR "SO-JIA" OR "SOJIA" OR "Still# disease" OR "Still# syndrome" OR (Enthesit* N5 arthrit*) OR (Enthesitis-related N5 arthrit*) OR "Spondyloarthr#path*" OR "JuSpA" OR "JSpA" OR "Ankylosing spondylitis" OR "JAS" OR (Psoria* N5 arthrit*) OR (Undifferentiated N5 arthrit*))) OR (AB ("Juvenile idiopathic arthrit*" OR "JIA" OR "Juvenile arthrit*" OR "JA" OR "Juvenile chronic arthrit*" OR "JCA" OR "Juvenile rheumatoid arthrit*" OR "JRA" OR "Juvenile rheumatic disease*" OR "Inflammatory arthropathy" OR "JRD" OR (Oligo* N5 arthrit*) OR "Oligoarthrit*" OR "oJIA" OR "OligoJIA" OR (Pauci* N5 arthrit*) OR (Poly* N5 arthrit*) OR (Systemic* N5 arthrit*) OR (Systemic-onset N5 arthrit*) OR "S-JIA" OR "SJIA" OR "SO-JIA" OR "SOJIA" OR "Still# disease" OR "Still# syndrome" OR (Enthesit* N5 arthrit*) OR (Enthesitis-related N5 arthrit*) OR "Spondyloarthr#path*" OR "JuSpA" OR "JSpA" OR "Ankylosing spondylitis" OR "JAS" OR (Psoria* N5 arthrit*) OR (Undifferentiated N5 arthrit*))) | **PsycINFO search format:**  (DE "Pain") OR (DE "Chronic pain") OR (DE "Acute pain") OR (DE "Injections") OR (DE "Intramuscular injections") OR (DE "Subcutaneous injections") OR (DE "Intravenous Injections") OR (DE "Pain perception") OR (DE "Pain thresholds") OR (DE "Pain management") OR (DE "Analgesia") OR (DE "Analgesic drugs") OR (DE "Pain measurement") OR (TI ("Pain*" OR "Hurt*" OR "Discomfort*" OR "Chronic pain" OR "Acute pain" OR "Procedural pain" OR "Needle*" OR "Injection*" OR "Syringe*" OR "Experimental pain" OR "Cold pressor" OR "Quantitative sensory test*" OR "Water load" OR "Heat pain" OR "Thermal pain" OR "Pressure pain" OR "Exercise task" OR "Nocicepti*" OR "Pain* threshold*" OR "Hyperalgesi*" OR "Hypoalgesi*" OR "Enthesalgi*" OR "Central sensitivity" OR "Somatosensory profile*" OR "Pain* management*" OR "Analgesi*" OR "Pain* measurement*")) OR (AB ("Pain*" OR "Hurt*" OR "Discomfort*" OR "Chronic pain" OR "Acute pain" OR "Procedural pain" OR "Needle*" OR "Injection*" OR "Syringe*" OR "Experimental pain" OR "Cold pressor" OR "Quantitative sensory test*" OR "Water load" OR "Heat pain" OR "Thermal pain" OR "Pressure pain" OR "Exercise task" OR "Nocicepti*" OR "Pain* threshold*" OR "Hyperalgesi*" OR "Hypoalgesi*" OR "Enthesalgi*" OR "Central sensitivity" OR "Somatosensory profile*" OR "Pain* management*" OR "Analgesi*" OR "Pain* measurement*")) | **PsycINFO search format:**  (DE "Child behavior") OR (DE "Child health") OR (DE "Pediatrics") OR (DE "Early Adolescence") OR (DE "Adolescent behavior") OR (DE "Adolescent health") OR (DE "Emerging Adulthood") OR (TI ("Infan*" OR "Perinat*" OR "Antepartum" OR "Ante-partum" OR "Postnatal*" OR "Post-natal*" OR "Baby*" OR "Babies" OR "Neonat*" OR "Neo-nat*" OR "Newborn*" OR "New-born*" OR "Child*" OR "Kid" OR "Kids" OR "Toddler*" OR "Girl*" OR "Girls" OR "Girlhood" OR "Boy*" OR "Boys" OR "Boyhood" OR "Preschool*" OR "Pre-school*" OR "Kindergarten*" OR "School*" OR "Juvenil*" OR "Minors*" OR "P?ediatric?" OR "Pediatric*" OR "Prepubescen*" OR "Pre-pubescen*" OR "Pubescen*" OR (Primary N2 school) OR (Primary N2 education) OR "Teen*" OR "Youth*" OR "Adolescen*" OR (Young N2 adult*) OR (Young N2 person*) OR (Young N2 individual*) OR (Young N2 people*) OR (Young N2 population*) OR "Student*" OR "Highschool*" OR "High-school*" OR (High N2 school*) OR (Secondary N2 school*))) OR (AB ("Infan*" OR "Perinat*" OR "Antepartum" OR "Ante-partum" OR "Postnatal*" OR "Post-natal*" OR "Baby*" OR "Babies" OR "Neonat*" OR "Neo-nat*" OR "Newborn*" OR "New-born*" OR "Child*" OR "Kid" OR "Kids" OR "Toddler*" OR "Girl*" OR "Girls" OR "Girlhood" OR "Boy*" OR "Boys" OR "Boyhood" OR "Preschool*" OR "Pre-school*" OR "Kindergarten*" OR "School*" OR "Juvenil*" OR "Minors*" OR "P?ediatric?" OR "Pediatric*" OR "Prepubescen*" OR "Pre-pubescen*" OR "Pubescen*" OR (Primary N2 school) OR (Primary N2 education) OR "Teen*" OR "Youth*" OR "Adolescen*" OR (Young N2 adult*) OR (Young N2 person*) OR (Young N2 individual*) OR (Young N2 people*) OR (Young N2 population*) OR "Student*" OR "Highschool*" OR "High-school*" OR (High N2 school*) OR (Secondary N2 school*))) |
| **Embase search format:**  ('Juvenile rheumatoid arthritis'/de) OR ('Polyarthritis'/de) OR ('Systemic juvenile idiopathic arthritis'/de) OR ('Enthesitis'/de) OR ('Spondyloarthropathy'/de) OR ('Spondylarthritis'/de) OR ('Ankylosing spondylitis'/de) OR ('Psoriatic arthritis'/de) OR ('Juvenile idiopathic arthrit*' OR 'JIA' OR 'Juvenile arthrit*' OR 'JA' OR 'Juvenile chronic arthrit*' OR 'JCA' OR 'Juvenile rheumatoid arthrit*' OR 'JRA' OR 'Juvenile rheumatic disease*' OR 'Inflammatory arthropathy' OR 'JRD' OR (Oligo* NEAR/5 arthrit*) OR 'Oligoarthrit*' OR 'oJIA' OR 'OligoJIA' OR (Pauci* NEAR/5 arthrit*) OR (Poly* NEAR/5 arthrit*) OR (Systemic* NEAR/5 arthrit*) OR (Systemic-onset NEAR/5 arthrit*) OR 'S-JIA' OR 'SJIA' OR 'SO-JIA' OR 'SOJIA' OR 'Still$ disease' OR 'Still$ syndrome' OR (Enthesit* NEAR/5 arthrit*) OR (Enthesitis-related NEAR/5 arthrit*) OR 'Spondyloarthr$path*' OR 'JuSpA' OR 'JSpA' OR 'Ankylosing spondylitis' OR 'JAS' OR (Psoria* NEAR/5 arthrit*) OR (Undifferentiated NEAR/5 arthrit*)):ti,ab,kw | **Embase search format:**  ('Pain'/de) OR ('Chronic pain'/de) OR ('Intractable pain'/de) OR ('Procedural pain'/de) OR ('Injection'/de) OR ('Intramuscular drug administration'/de) OR ('Intraarticular drug administration'/de) OR ('Subcutaneous drug administration'/de) OR ('Intravenous drug administration'/de) OR ('Syringe'/de) OR ('Nociception'/de) OR ('Pain threshold'/de) OR ('Analgesia'/de) OR ('Analgesics'/de) OR ('Pain measurement'/de) OR ('Pain*' OR 'Hurt*' OR 'Discomfort*' OR 'Chronic pain' OR 'Acute pain' OR 'Procedural pain' OR 'Needle*' OR 'Injection*' OR 'Syringe*' OR 'Experimental pain' OR 'Cold pressor' OR 'Quantitative sensory test*' OR 'Water load' OR 'Heat pain' OR 'Thermal pain' OR 'Pressure pain' OR 'Exercise task' OR 'Nocicepti*' OR 'Pain* threshold*' OR 'Hyperalgesi*' OR 'Hypoalgesi*' OR 'Enthesalgi*' OR 'Central sensitivity' OR 'Somatosensory profile*' OR 'Pain* management*' OR 'Analgesi*' OR 'Pain* measurement'):ti,ab,kw | **Embase search format:**  ('Infant'/exp) OR ('Newborn'/exp) OR ('Child'/exp) OR ('Preschool child'/exp) OR ('Child behavior'/exp) OR ('Child health'/exp) OR ('Pediatrics'/exp) OR ('Adolescent'/exp) OR ('Adolescence'/exp) OR ('Adolescent behavior'/exp) OR ('Adolescent health'/exp) OR ('Young adult'/exp) OR ('Infan*' OR 'Perinat*' OR 'Antepartum' OR 'Ante-partum' OR 'Postnatal*' OR 'Post-natal*' OR 'Baby*' OR 'Babies' OR 'Neonat*' OR 'Neo-nat*' OR 'Newborn*' OR 'New-born*' OR 'Child*' OR 'Kid' OR 'Kids' OR 'Toddler*' OR 'Girl*' OR 'Girls' OR 'Girlhood' OR 'Boy*' OR 'Boys' OR 'Boyhood' OR 'Preschool*' OR 'Pre-school*' OR 'Kindergarten*' OR 'School*' OR 'Juvenil*' OR 'Minors*' OR 'P$ediatric$' OR 'Pediatric*' OR 'Prepubescen*' OR 'Pre-pubescen*' OR 'Pubescen*' OR (Primary NEAR/2 school) OR (Primary NEAR/2 education) OR 'Teen*' OR 'Youth*' OR 'Adolescen*' OR (Young NEAR/2 adult*) OR (Young NEAR/2 person*) OR (Young NEAR/2 individual*) OR (Young NEAR/2 people*) OR (Young NEAR/2 population*) OR 'Student*' OR 'Highschool*' OR 'High-school*' OR (High NEAR/2 school*) OR (Secondary NEAR/2 school*)):ti,ab,kw |
| **Scopus search format:**  TITLE-ABS-KEY ("Juvenile idiopathic arthrit*" OR "JIA" OR "Juvenile arthrit*" OR "JA" OR "Juvenile chronic arthrit*" OR "JCA" OR "Juvenile rheumatoid arthrit*" OR "JRA" OR "Juvenile rheumatic disease*" OR "Inflammatory arthropathy" OR "JRD" OR (Oligo* W/5 arthrit*) OR "Oligoarthrit*" OR "oJIA" OR "OligoJIA" OR (Pauci* W/5 arthrit*) OR (Poly* W/5 arthrit*) OR (Systemic* W/5 arthrit*) OR (Systemic-onset W/5 arthrit*) OR "S-JIA" OR "SJIA" OR "SO-JIA" OR "SOJIA" OR "Still? disease" OR "Still? syndrome" OR (Enthesit* W/5 arthrit*) OR (Enthesitis-related W/5 arthrit*) OR "Spondyloarthr?path*" OR "JuSpA" OR "JSpA" OR "Ankylosing spondylitis" OR "JAS" OR (Psoria* W/5 arthrit*) OR (Undifferentiated W/5 arthrit*)) | **Scopus search format:**  TITLE-ABS-KEY ("Pain*" OR "Hurt*" OR "Discomfort*" OR "Chronic pain" OR "Acute pain" OR "Procedural pain" OR "Needle*" OR "Injection*" OR "Syringe*" OR "Experimental pain" OR "Cold pressor" OR "Quantitative sensory test*" OR "Water load" OR "Heat pain" OR "Thermal pain" OR "Pressure pain" OR "Exercise task" OR "Nocicepti*" OR "Pain* threshold*" OR "Hyperalgesi*" OR "Hypoalgesi*" OR "Enthesalgi*" OR "Central sensitivity" OR "Somatosensory profile*" OR "Pain* management*" OR "Analgesi*" OR "Pain* measurement*”) | **Scopus search format:**  TITLE-ABS-KEY ("Infan*" OR "Perinat*" OR "Antepartum" OR "Ante-partum" OR "Postnatal*" OR "Post-natal*" OR "Baby*" OR "Babies" OR "Neonat*" OR "Neo-nat*" OR "Newborn*" OR "New-born*" OR "Child*" OR "Kid" OR "Kids" OR "Toddler*" OR "Girl*" OR "Girls" OR "Girlhood" OR "Boy*" OR "Boys" OR "Boyhood" OR "Preschool*" OR "Pre-school*" OR "Kindergarten*" OR "School*" OR "Juvenil*" OR "Minors*" OR "P?ediatric?" OR "Pediatric*" OR "Prepubescen*" OR "Pre-pubescen*" OR "Pubescen*" OR (Primary W/2 school) OR (Primary W/2 education) OR "Teen*" OR "Youth*" OR "Adolescen*" OR (Young W/2 adult*) OR (Young W/2 person*) OR (Young W/2 individual*) OR (Young W/2 people*) OR (Young W/2 population*) OR "Student*" OR "Highschool*" OR "High-school*" OR (High W/2 school*) OR (Secondary W/2 school*)) |
| **Cochrane search format:**  "Arthritis, juvenile" [MeSH] OR "Spondyloarthropathies" [MeSH] OR "Spondylitis, Ankylosing" [MeSH] OR "Spondylarthritis" [MeSH] OR "Arthritis, psoriatic" [MeSH] OR ("Juvenile idiopathic arthrit*" OR "JIA" OR "Juvenile arthrit*" OR "JA" OR "Juvenile chronic arthrit*" OR "JCA" OR "Juvenile rheumatoid arthrit*" OR "JRA" OR "Juvenile rheumatic disease*" OR "Inflammatory arthropathy" OR "JRD" OR (Oligo* near/5 arthrit*) OR "Oligoarthrit*" OR "oJIA" OR "OligoJIA" OR (Pauci* near/5 arthrit*) OR (Poly* near/5 arthrit*) OR (Systemic* near/5 arthrit*) OR (Systemic-onset near/5 arthrit*) OR "S-JIA" OR "SJIA" OR "SO-JIA" OR "SOJIA" OR "Still? disease" OR "Still? syndrome" OR (Enthesit* near/5 arthrit*) OR (Enthesitis-related near/5 arthrit*) OR "Spondyloarthr?path*" OR "JuSpA" OR "JSpA" OR "Ankylosing spondylitis" OR "JAS" OR (Psoria* near/5 arthrit*) OR (Undifferentiated near/5 arthrit*)):ti,ab,kw | **Cochrane search format:**  "Pain" [MeSH] OR "Chronic pain" [MeSH] OR "Intractable pain" [MeSH] OR "Acute pain" [MeSH] OR "Pain, procedural" [MeSH] OR "Injections" [MeSH] OR "Injections, intramuscular" [MeSH] OR "Injections, intra-articular" [MeSH] OR "Injections, subcutaneous" [MeSH] OR "Injections, intravenous' [MeSH] OR "Syringes" [MeSH] OR "Pain perception" [MeSH] OR "Nociceptive pain" [MeSH] OR "Pain management" [MeSH] OR "Analgesia" [MeSH] OR "Analgesics" [MeSH] OR "Pain measurement" [MeSH] OR OR "Pain threshold" [MeSH] OR (“Pain*" OR "Hurt*" OR "Discomfort*" OR "Chronic pain" OR "Acute pain" OR "Procedural pain" OR "Needle*" OR "Injection*" OR "Syringe*" OR "Experimental pain" OR "Cold pressor" OR "Quantitative sensory test*" OR "Water load" OR "Heat pain" OR "Thermal pain" OR "Pressure pain" OR "Exercise task" OR "Nocicepti*" OR "Pain* threshold*" OR "Hyperalgesi*" OR "Hypoalgesi*" OR "Enthesalgi*" OR "Central sensitivity" OR "Somatosensory profile*" OR "Pain* management*" OR "Analgesi*" OR "Pain* measurement*"):ti,ab,kw | **Cochrane search format:**  "Infant" [MeSH][exp] OR "Infant, Newborn" [MeSH][exp] OR "Infant behavior" [MeSH][exp] OR "Infant Health" [MeSH][exp] OR "Child" [MeSH][exp] OR "Child, preschool" [MeSH][exp] OR "Child behavior" [MeSH][exp] OR "Child health" [MeSH][exp] OR "Pediatrics" [MeSH][exp] OR "Adolescent" [MeSH] OR "Adolescent behavior" [MeSH][exp] OR "Adolescent health" [MeSH][exp] OR "Young adult" [MeSH][exp] OR ("Infan*" OR "Perinat*" OR "Antepartum" OR "Ante-partum" OR "Postnatal*" OR "Post-natal*" OR "Baby*" OR "Babies" OR "Neonat*" OR "Neo-nat*" OR "Newborn*" OR "New-born*" OR "Child*" OR "Kid" OR "Kids" OR "Toddler*" OR "Girl*" OR "Girls" OR "Girlhood" OR "Boy*" OR "Boys" OR "Boyhood" OR "Preschool*" OR "Pre-school*" OR "Kindergarten*" OR "School*" OR "Juvenil*" OR "Minors*" OR "P?ediatric?" OR "Pediatric*" OR "Prepubescen*" OR "Pre-pubescen*" OR "Pubescen*" OR (Primary near/2 school) OR (Primary near/2 education) OR "Teen*" OR "Youth*" OR "Adolescen*" OR (Young near/2 adult*) OR (Young near/2 person*) OR (Young near/2 individual*) OR (Young near/2 people*) OR (Young near/2 population*) OR "Student*" OR "Highschool*" OR "High-school*" OR (High near/2 school*) OR (Secondary near/2 school*)):ti,ab,kw |
