# Supplementary material for: A systematic review of the psychosocial factors associated with pain in children with juvenile idiopathic arthritis
Source: Pediatr Rheumatol Online J. 2023 Jun 16;21:57. doi: 10.1186/s12969-023-00828-5 (PMC10273767; doi:10.1186/s12969-023-00828-5)
Supplement: Supplementary file 2 — Additional file 2. Data Extraction Template, Template of information extracted from included articles. [file 12969_2023_828_MOESM2_ESM.docx]

Additional File 2: Data Extraction Template

| **Name of Data Extractor** |  |
| --- | --- |
| **Study Details** | - **Covidence study ID:** - **Full article title:** - **Journal:** - **First Author:** Last Name, First Name - **Year of publication:** - **Country of first author:** - **Publication type:** published article, abstract, dissertation, other (specify) - **Possible conflicts of interest:** yes (specify), no |
| **Study Population** | - **Sample size:** - **Sample:** youth, caregivers, healthcare providers, other (specify) - **For each population, fill out the relevant details below:**    - Age     - **Measurement:** Mean or median     - **Age:**     - **Age range:** - **Sex (percentage male, female, other):** - **Diagnosis (percentage polyarticular, oligoarticular, enthesitis, systemic, psoriatic, undifferentiated, other):** - **Disease status (percentage active, inactive, in remission):** - Length of the disease - **Measurement:** Disease duration or disease onset - **Measurement:** Mean or median - **Duration:** - **Range:** - **Other details:** |
| **Study Design** | - **Design:** cross-sectional, cohort, case control, case series, randomized control trial, quasi-experimental trial, other (specify) - **Start date:** - **End date:** - **Study Duration/Follow-Up:** - **Countries recruited from:** - **Setting:** clinics, community, other (specify) - **Was this sample from a cohort:** no, yes (specify) - **Other details:** |
| **Measures** | - Exposure (i.e., psychosocial factor) - **Construct:** - **Data:** numerical or categorial - **Reporter:** youth, parent, healthcare provider, other (specify) - **Measure:** - **Citation:** - Outcome (i.e., pain) - **Measurement**: intensity, frequency, or sensitivity - **Data:** numerical or categorical - **Reporter:** youth, parent, healthcare provider, other (specify) - **Measure:** - **Time (e.g., current, past week):** - **Citation:** |
| **Results** | - Association (complete the following for each) - **Exposure (i.e., psychosocial factor)** - **Outcome (i.e., pain)** - **Significance of association:** yes or no - **Direction of association:** positive or negative - **Other information (e.g., statistical technique, covariates):** |
