# Supplementary material for: A systematic review of the psychosocial factors associated with pain in children with juvenile idiopathic arthritis
Source: Pediatr Rheumatol Online J. 2023 Jun 16;21:57. doi: 10.1186/s12969-023-00828-5 (PMC10273767; doi:10.1186/s12969-023-00828-5)
Supplement: Supplementary file 3 — Additional file 3: 3.1. Quasi-Experimental Studies, Results and Discussion of Quasi-Experimental Studies. 3.2. Critical Appraisal Results for Quasi-Experimental Studies, Critical Appraisal Results Table for Quasi-Experimental Studies. 3.3. Quasi-Experimental Study Characteristics and Results, Study Characteristics and Results Table for Quasi-Experimental Studies. [file 12969_2023_828_MOESM3_ESM.docx]

Additional File 3.1: Quasi-Experimental Studies

**Methodological Quality**

For the five quasi-experimental studies, critical appraisal scores ranged from 78% to 100%, with the lack of a comparison group and participant retention as biggest limitations (Additional File 3.2).

**Results**

***Child factors.*** Across five psychosocial interventions, which may be considered as an external source of support in completing a secondary appraisal, 19/24 associations were significant [61,63,77,78,90]. Walco [90] tested the efficacy of an 8-week cognitive-behavioral therapy (CBT) intervention in 13 children with JIA and their parents and followed up six and 12 months later. All 10 of the associations demonstrated reduced pain intensity post-intervention. Comparatively, Lomholt and colleagues [63] ran a 6-week group-based CBT intervention for nine children with JIA. After the intervention; however, no differences in pain intensity were observed between the treatment and control groups. Lavigne and colleagues [61] provided a 6-week treatment package for pain management to eight children with JIA and their parents. Despite the small sample size, 6/9 associations demonstrated the benefits of the intervention in reducing both pain intensity and frequency, and the remaining were trending in the expected direction. Stinson and colleagues [77] developed the iPeer2Peer intervention and assessed the efficacy of an 8-week trial in 16 children with JIA and a control group. No differences emerged in pain intensity scores between the treatment and control group post intervention. Stinson and colleagues [78] also developed the 12-week Teens Taking Charge intervention and compared the outcomes at 3, 6, and 12 months for 88 children who participated in the intervention to 131 controls. All three associations showed significantly reduced pain intensity in the treatment condition at all timepoints. Taken together, participation in CBT or pain specific interventions tends to be predictive of lower pain reports in children with JIA.

**Discussion**

The efficacy of five psychosocial interventions varying in their orientation and delivery in reducing JIA pain were reviewed. Most demonstrated significant reductions in JIA pain intensity and frequency post intervention or in comparison to the control group. While a complete review and comparison of these interventions is beyond the scope of this study, Cohen [28] and Butler [27] have recently published comprehensive reviews in this area. Nevertheless, psychosocial interventions are promising way to foster improvements in JIA pain along with other important outcomes.

| Additional File 3.2. Critical Appraisal Results for Quasi-Experimental Studies | | | | | | | | | | |
| --- | --- | --- | --- | --- | --- | --- | --- | --- | --- | --- |
| **Author & Year** | **Q1** | **Q2** | **Q3** | **Q4** | **Q5** | **Q6** | **Q7** | **Q8** | **Q9** | **%** |
| Lavigne 1992 [61] | Y | U | Y | Y | Y | Y | Y | Y | Y | 89% |
| Lomholt 2015 [63] | Y | Y | Y | Y | Y | Y | Y | Y | Y | 100% |
| Stinson 2016 [77] | Y | U | Y | Y | Y | Y | Y | Y | Y | 89% |
| Stinson 2020 [78] | Y | U | Y | Y | Y | N | Y | Y | Y | 78% |
| Walco 1992 [90] | Y | Y | Y | N | Y | N | Y | Y | Y | 78% |
| % | 100% | 40% | 100% | 80% | 100% | 60% | 100% | 100% | 100% |  |
| ^‡, ‡‡, ‡‡‡, †, ††, §, §§^ Studies with overlapping datasets  Y = Yes; N = No; U = Unclear  JBI critical appraisal for quasi-experimental studies: Q1 = Is it clear in the study what is the ‘cause’ and what is the ‘effect’ (i.e., there is no confusion about which variable comes first)? Q2 = Were the participants included in any comparisons similar? Q3 = Were the participants included in any comparisons receiving similar treatment/care, other than the exposure or intervention of interest? Q4 = Was there a control group? Q5 = Were there multiple measurements of the outcome both pre and post the intervention/exposure? Q6 = Was follow up complete/were differences between groups in terms of their follow up adequately described and analyzed? Q7 = Were the outcomes of participants included in any comparisons measured in the same way? Q8 = Were outcomes measured in a reliable way? Q9 = Was appropriate statistical analysis used? | | | | | | | | | | |

| Additional File 3.3. Quasi-Experimental Study Characteristics and Results | | | | | | | | |
| --- | --- | --- | --- | --- | --- | --- | --- | --- |
| **Author,**  **Year, Publication Type** | **Sample Size(s)** | **Age(s) x̄ or x͂ (Range)** | **% Female** | **% JIA Type** | **Pain: Construct (Reporter) – Measure** | **Psychosocial Factor(s): Construct (Reporter) – Measure** | **Main Findings: Analysis - Result** | |
| Lavigne  1992 [61]  Article | 8 C 7 P 5 HCP | Mdn=14 (9-17)  -- -- | 88 100 -- | Po:75; O:13; E:13 | PI (C & P) – VAS 3x/day for 1 mos pre, post, and 6 mos later  PF (C & P) – VAS the percentage of ratings above 5  PS (HCP) – -- | Treatment (C) – 6 sessions of biweekly therapy for pain management / Waitlist Control | Mann Whitney U-Test –  Treatment and Waitlist Control groups did not significantly differ in child reported PI and PF  The treatment group had significantly lower parent reported PI and PF compared to Waitlist Control  Repeated Measured ANOVA –  Children and parents reported PI and PF, and HCP reported PS tended to decrease over time in response to the treatment | |
| Lomholt  2015 [63]  Article | 19 C | * (9-14) | 79 | Po:32; O:42; E:5; S:11; Ps:11 | PI (C) – FPS-R assessed 2x/day for 1 week (averaged) | Treatment (C) – 6 sessions of Cognitive Behavioral Therapy group/Waitlist Control | ANCOVA controlling for pre-intervention data and disease status – Treatment and Waitlist Control groups did not significantly differ in PI post treatment | |
| Stinson  2016 [77]  Article | 32 C | x̄=14 (12-17) | 97 | Po:41; O:31; E:3; Ps:25 | PI (C) – NRS RPI assessed at baseline and post intervention | Treatment (C) – iPeer2Peer intervention for 8 weeks / WLC | Marginal Linear Models – Treatment and WLC groups did not significantly differ in their PI at study completion | |
| Stinson  2020 [78]  Article | 219 C 197 P | x̄=14 (12-17)  -- | 70 80 | Po:32; O:32; E:16; S:2; Ps:11; U:7 | PI (C) – NRS RPI assessed at baseline, 3 mos (post intervention), 6 mos, and 12 mos | Treatment (C) – Teens Taking Charge intervention for 12 weeks / WLC | LMM – The treatment group demonstrated significantly lower PI at 3, 6, and 12 mos compared to the WLC | |
| Walco  1992 [90]  Article | 13 C 13 P | x̄=10 (4-16)  -- | 62 -- | O:62; S:38 | PI (C & P) – VAS PPQ assessed 2x/day at baseline, post intervention, 6 and 12 mos | Treatment (C) – 8 Week Cognitive Behavioral Therapy Intervention | T-test – Child and parent reported PI (AM and PM) significantly decreased from baseline to post intervention, 6 mos, and 12 mos | |
| Underlined text represents significant results.  ANOVA = Analysis of Variance; ANCOVA = Analysis of Covariance; C = Child; E = Enthesitis-Related Arthritis; HCP = Healthcare providers; LMM = Linear Mixed Models; O = Oligoarticular Arthritis; P = Parents/Caregivers; Po = Polyarticular Arthritis; Ps = Psoriatic Arthritis; PF = Pain frequency; PI = Pain intensity; S = Systemic Arthritis; U = Undifferentiated/Other Arthritis | | | | | | | |  |
