# Supplementary material for: A systematic review of the psychosocial factors associated with pain in children with juvenile idiopathic arthritis
Source: Pediatr Rheumatol Online J. 2023 Jun 16;21:57. doi: 10.1186/s12969-023-00828-5 (PMC10273767; doi:10.1186/s12969-023-00828-5)
Supplement: Supplementary file 4 — Additional file 4. Summary of Results, Summary of results in tabular form. [file 12969_2023_828_MOESM4_ESM.docx]

Additional File 4: Summary of Results

| Summary of Psychosocial Correlates with Pain Intensity, Frequency, and Sensitivity | | | | | | | | | | | | | | | | | | | | | | | | | | | | | | | | | | | | | | | | | | | | | | | | | | | | | | | | | | | | | | |
| --- | --- | --- | --- | --- | --- | --- | --- | --- | --- | --- | --- | --- | --- | --- | --- | --- | --- | --- | --- | --- | --- | --- | --- | --- | --- | --- | --- | --- | --- | --- | --- | --- | --- | --- | --- | --- | --- | --- | --- | --- | --- | --- | --- | --- | --- | --- | --- | --- | --- | --- | --- | --- | --- | --- | --- | --- | --- | --- | --- | --- | --- | --- |
|  | | Amine 2009 [38] | Anthony 2011^‡^ [39] | Armbrust 2016 [40] | Baildam 1995 [41] | Baloueff 1996 [42] | Barlow 2000 [43] | Barlow 2001 [44] | Barlow 2002 [45] | Bromberg 2009^‡^ [46] | Bromberg 2012^‡^ [47] | Bruns 2008 [48] | Connelly 2012 [49] | Cornelissen 2014 [50] | Dimitrijevic C. 2019 [51] | Doherty 1993 [52] | El-Najjar 2014 [53] | Hagglund 1995 [54] | Hanns 2018-1^‡‡^ [55] | Hanns 2018-2^‡‡^ [55] | Hoff 2006 [56] | Jaworski 1992 [57] | Klotsche 2014 [58] | Kovalchuk 2017 [59] | Kovalchuk 2018 [60] | Lavigne 1992 [61] | Listing 2018 [62] | Lomholt 2013^††^ [64] | Lomholt 2015 [63] | Luca 2017 [65] | Mahler 2017 [66] | Margetić 2005 [67] | Oen 2009^§^ [69] | Oen 2021^§^ [68] | Rashid 2018^‡‡^ [4] | Ross 1993 [70] | Sällfors 2004 [71] | Schanberg 2003‡ [2] | Schanberg 2005‡ [72] | Selvaag 2003 [73] | Selvaag 2005 [74] | Shelepina 2011 [75] | Stinson 2006-1^†^ [76] | Stinson 2006-2 [76] | Stinson 2016 [77] | Stinson 2020 [78] | Tarakci 2011 [79] | Tarkiainen 2019 [80] | Thastum 1997 [81] | Thastum 1998 [82] | Thastum 2005^††^ [25] | Thastum 2011^††^ [83] | Thompson 1987^‡‡‡^ [26] | Tupper 2012 [84] | Tupper 2013^†^ [3] | Upadhyay 2021 [85] | Vandvik 1990 [86] | Vuorimaa 2008^§§^ [89] | Vuorimaa 2009^§§^ [88] | Vuorimaa 2011^§§^ [87] | Walco 1992 [90] | Yan 2020 [91] |
| Child Correlates | |  |  |  |  |  |  |  |  |  |  |  |  |  |  |  |  |  |  |  |  |  |  |  |  |  |  |  |  |  |  |  |  |  |  |  |  |  |  |  |  |  |  |  |  |  |  |  |  |  |  |  |  |  |  |  |  |  |  |  |  |  |
|  | Mental Health |  |  |  | **/** |  |  |  |  |  |  |  |  | **/** |  |  |  |  |  |  |  |  | **-/** |  | **/** |  |  |  |  |  |  |  |  |  |  |  |  |  |  |  |  |  |  |  |  |  |  |  |  |  |  |  | **/** |  |  |  | **/** |  |  |  |  |  |
|  | Externalizing Symptoms |  |  |  |  | **/** |  |  |  |  |  |  |  |  |  |  |  |  |  |  |  |  |  |  | **/** |  |  |  |  |  |  |  |  |  |  | **/** |  |  |  |  |  |  |  |  |  |  |  |  |  |  |  |  | **/** |  |  |  | **/** |  |  |  |  |  |
|  | Internalizing Symptoms |  | **/** |  |  |  |  |  |  |  |  |  |  |  | **+** |  |  |  |  |  |  |  |  |  |  |  |  |  |  |  |  |  |  |  |  | **+** |  |  |  |  |  |  |  |  |  |  |  |  |  |  |  |  | **/** |  |  |  | **/** | **+** | **+** |  |  |  |
|  | Anxiety Symptoms |  |  |  |  |  |  |  |  |  |  |  |  | **+/** |  |  |  |  |  |  |  |  |  |  |  |  |  |  |  |  |  | **/** |  |  |  | **+** |  | **++** |  |  |  |  | **+** | **/** |  |  | **/** |  |  |  |  |  |  |  |  | **/** |  |  |  | **+** |  |  |
|  | Depression Symptoms |  |  |  |  |  |  |  |  |  | **+/** |  | **++//** |  |  |  | **+** | **/** | **++** | **+/** | **+/** | **+/** |  |  |  |  |  |  |  |  |  | **+** |  |  | **++/** | **+/** |  | **/** | **+** |  |  |  |  |  |  |  | **/** |  |  |  |  |  |  | **+** |  | **/** |  |  |  | **+** |  | **+** |
|  | Daily Interference |  |  |  |  |  |  |  |  |  |  |  |  |  |  |  |  |  |  |  |  |  |  |  | **+** |  |  |  |  |  |  |  |  |  |  |  |  |  |  |  |  |  | **+** | **+** |  |  |  |  |  |  |  |  |  |  |  | **/** |  |  |  |  |  |  |
|  | Pain Beliefs - Disability |  |  |  |  |  |  |  |  |  |  |  |  |  |  |  |  |  |  |  |  |  |  |  |  |  |  | **+** |  |  |  |  |  |  |  |  |  |  |  |  |  |  |  |  |  |  |  |  |  |  | **+** | **++** |  |  |  |  |  |  |  |  |  |  |
|  | Pain Beliefs - Harm |  |  |  |  |  |  |  |  |  |  |  |  |  |  |  |  |  |  |  |  |  |  |  |  |  |  | **+** |  |  |  |  |  |  |  |  |  |  |  |  |  |  |  |  |  |  |  |  |  |  | **+** | **++** |  |  |  |  |  |  |  |  |  |  |
|  | Pain Beliefs - Emotion & Solicitude |  |  |  |  |  |  |  |  |  |  |  |  |  |  |  |  |  |  |  |  |  |  |  |  |  |  |  |  |  |  |  |  |  |  |  |  |  |  |  |  |  |  |  |  |  |  |  |  |  | **+/** |  |  |  |  |  |  |  |  |  |  |  |
|  | Pain Beliefs - No Control |  |  |  |  |  |  |  |  |  |  |  |  |  |  |  |  |  |  |  |  |  |  |  |  |  |  | **/** |  |  |  |  |  |  |  |  |  |  |  |  |  |  |  |  |  |  |  |  |  |  | **+** | **++** |  |  |  |  |  |  |  |  |  |  |
|  | Pain Beliefs - Medical Cure |  |  |  |  |  |  |  |  |  |  |  |  |  |  |  |  |  |  |  |  |  |  |  |  |  |  |  |  |  |  |  |  |  |  |  |  |  |  |  |  |  |  |  |  |  |  |  |  |  | **+/** | **//** |  |  |  |  |  |  |  |  |  |  |
|  | Self-Esteem |  |  |  |  | **/** |  |  |  |  |  |  |  |  |  |  |  |  |  |  |  |  |  |  | **/** |  |  |  |  |  |  |  |  |  |  |  |  |  |  |  |  |  |  |  |  |  |  |  |  |  |  |  |  |  |  |  |  |  |  |  |  |  |
|  | Self-Efficacy - Activity |  |  |  |  |  |  | **-** |  |  |  |  |  |  |  |  |  |  |  |  |  |  |  |  |  |  |  |  |  |  |  |  |  |  |  |  |  |  |  |  |  |  |  |  |  |  |  |  |  |  |  |  |  |  |  |  |  |  |  |  |  |  |
|  | Self-Efficacy - Psychological |  |  |  |  |  |  | **-** |  |  |  |  |  |  |  |  |  |  |  |  |  |  |  |  |  |  |  |  |  |  |  |  |  |  |  |  |  |  |  |  |  |  |  |  |  |  |  |  |  |  |  |  |  |  |  |  |  |  |  | **/** |  |  |
|  | Self-Efficacy - Social |  |  |  |  |  |  |  |  |  |  |  |  |  |  |  |  |  |  |  |  |  |  |  |  |  |  |  |  |  |  |  |  |  |  |  |  |  |  |  |  |  |  |  |  |  |  |  |  |  |  |  |  |  |  |  |  |  |  | **-** |  |  |
|  | Self-Efficacy - Symptom |  |  |  |  |  |  | **-** |  |  |  |  |  |  |  |  |  |  |  |  |  |  |  |  |  |  |  |  |  |  |  |  |  |  |  |  |  |  |  |  |  |  |  |  |  |  |  |  |  |  |  |  |  |  |  |  |  |  |  | **/** |  |  |
|  | Coping Efficacy |  |  |  |  |  |  |  |  | **-** |  |  |  |  |  |  |  |  |  |  |  |  |  |  |  |  |  |  |  |  |  |  |  |  |  |  |  |  |  |  |  |  |  |  |  |  |  |  |  |  |  |  |  |  |  |  |  |  |  | **-** |  |  |
|  | Coping - Distraction |  |  |  |  |  |  |  |  |  |  |  |  |  |  |  |  |  |  |  |  |  |  |  |  |  |  | **/** |  |  |  |  |  |  |  |  |  |  |  |  |  |  | **/** | **/** |  |  |  |  | **//** | **-/** | **//** |  |  |  |  |  |  |  |  |  |  |  |
|  | Coping - Catastrophizing |  |  |  |  |  |  |  |  |  |  |  |  | **/** | **+** |  |  |  |  |  |  |  |  |  |  |  |  | **+** |  |  |  |  |  |  |  |  |  |  |  |  |  |  |  |  |  |  |  |  | **++/** | **+/** | **+//** |  |  |  |  |  |  |  |  |  |  |  |
|  | Coping - Externalizing |  |  |  |  |  |  |  |  |  |  |  |  |  |  |  |  |  |  |  |  |  |  |  |  |  |  |  |  |  |  |  |  |  |  |  |  |  |  |  |  |  |  |  |  |  |  |  |  | **/** | **/-** |  |  |  |  |  |  |  |  |  |  |  |
|  | Coping - Information Seeking |  |  |  |  |  |  |  |  |  |  |  |  |  |  |  |  |  |  |  |  |  |  |  |  |  |  |  |  |  |  |  |  |  |  |  |  |  |  |  |  |  |  |  |  |  |  |  |  | **/** | **/** |  |  |  |  |  |  |  |  |  |  |  |
|  | Coping - Seeking Social Support |  |  |  |  |  |  |  |  |  |  |  |  |  |  |  |  |  |  |  |  |  |  |  |  |  |  |  |  |  |  |  |  |  |  |  |  |  |  |  |  |  |  |  |  |  |  |  |  | **/** | **//** |  |  |  |  |  |  |  |  |  |  |  |
|  | Coping - Positive Self-Statements |  |  |  |  |  |  |  |  |  |  |  |  |  |  |  |  |  |  |  |  |  |  |  |  |  |  | **/** |  |  |  |  |  |  |  |  |  |  |  |  |  |  |  |  |  |  |  |  |  | **-/** | **-//** |  |  |  |  |  |  |  |  |  |  |  |
|  | Coping - Reinterpretation |  |  |  |  |  |  |  |  |  |  |  |  |  |  |  |  |  |  |  |  |  |  |  |  |  |  |  |  |  |  |  |  |  |  |  |  |  |  |  |  |  |  |  |  |  |  |  | **//** |  |  |  |  |  |  |  |  |  |  |  |  |  |
|  | Coping - Approach |  |  |  |  |  |  |  |  |  |  |  |  |  |  |  |  |  |  |  |  |  |  |  |  |  |  |  |  |  |  |  |  |  |  |  |  |  |  |  |  |  | **/** | **/** |  |  |  |  |  |  |  |  |  |  |  |  |  |  |  |  |  |  |
|  | Coping - Emotion Focused Avoidance |  |  |  |  |  |  |  |  |  |  |  |  |  |  |  |  |  |  |  |  |  |  |  |  |  |  |  |  |  |  |  |  |  |  |  |  |  |  |  |  |  | **+/** | **/** |  |  |  |  |  |  |  |  |  |  |  |  |  |  |  |  |  |  |
|  | School Functioning |  |  | **-** |  | **/** |  |  |  |  |  |  |  |  |  | **-/** |  |  |  |  |  |  | **-/** |  |  |  |  |  |  |  |  |  |  |  |  |  | **--** |  | **/** |  |  | **-** |  |  |  |  |  |  |  |  |  |  |  |  |  | **-/** |  |  |  |  |  |  |
|  | Social Functioning |  |  |  |  | **/** |  |  |  |  |  |  |  |  |  |  |  | **/** |  |  |  |  | **-/** |  |  |  |  |  |  |  |  |  |  |  |  |  |  | **-** | **-** |  |  |  | **/** | **/** |  |  |  |  |  |  |  |  | **/** |  |  |  |  |  |  |  |  |  |
|  | Quality of Life | **-** |  |  |  |  |  |  |  |  |  |  |  |  |  |  |  |  |  |  |  |  | **-** | **/** | **/** |  | **-** |  |  | **/** |  |  | **-** | **-/** |  |  |  |  |  | **/** |  |  | **-** | **-/** |  |  |  | **-** |  |  |  |  |  |  | **-** |  | **/** |  |  |  |  |  |
|  | Well-being |  |  |  |  |  |  |  |  |  |  |  |  |  |  |  |  |  |  |  |  |  |  |  | **-** |  |  |  |  |  | **/** |  | **-** |  | **--/** |  | **--** |  |  |  | **-** |  |  |  |  |  | **-** |  |  |  |  |  |  |  |  |  |  |  |  | **-** |  |  |
|  | Intervention Participation |  |  |  |  |  |  |  |  |  |  |  |  |  |  |  |  |  |  |  |  |  |  |  |  | **-/-//** |  |  | **/** |  |  |  |  |  |  |  |  |  |  |  |  |  |  |  | **/** | **-** |  |  |  |  |  |  |  |  |  |  |  |  |  |  | **-** |  |
| Parent Correlates | |  |  |  |  |  |  |  |  |  |  |  |  |  |  |  |  |  |  |  |  |  |  |  |  |  |  |  |  |  |  |  |  |  |  |  |  |  |  |  |  |  |  |  |  |  |  |  |  |  |  |  |  |  |  |  |  |  |  |  |  |  |
|  | Internalizing Symptoms |  | **/** |  |  |  |  |  | **/** |  |  | **/** |  |  |  |  |  |  |  |  |  |  |  |  | **+/** |  |  |  |  |  |  |  |  |  |  | **+** |  |  |  |  |  |  |  |  |  |  |  |  |  |  |  |  |  |  |  |  |  |  |  | **+/** |  |  |
|  | Daily Interference |  | **-/** |  |  |  |  |  |  |  |  | **/** |  |  |  |  |  |  |  |  |  |  |  |  | **+/** |  |  |  |  |  |  |  |  |  |  |  |  |  |  |  |  |  |  |  |  |  |  |  |  |  |  |  |  |  |  |  |  |  |  |  |  |  |
|  | Self-Esteem |  |  |  |  |  |  |  |  |  |  |  |  |  |  |  |  |  |  |  |  |  |  |  |  |  |  |  |  |  |  |  |  |  |  |  |  |  |  |  |  |  |  |  |  |  |  |  |  |  |  |  |  |  |  |  |  |  |  |  |  |  |
|  | Self-Efficacy - Psychosocial |  |  |  |  |  | **-/** |  | **/** |  |  |  |  |  |  |  |  |  |  |  |  |  |  |  |  |  |  |  |  |  |  |  |  |  |  |  |  |  |  |  |  |  |  |  |  |  |  |  |  |  |  |  |  |  |  |  |  |  |  | **-** |  |  |
|  | Self-Efficacy - Psychological |  |  |  |  |  |  |  |  |  |  |  |  |  |  |  |  |  |  |  |  |  |  |  |  |  |  |  |  |  |  |  |  |  |  |  |  |  |  |  |  |  |  |  |  |  |  |  |  |  |  |  |  |  |  |  |  |  |  | **/** |  |  |
|  | Self-Efficacy - Symptom |  |  |  |  |  | **-/** |  | **/** |  |  |  |  |  |  |  |  |  |  |  |  |  |  |  |  |  |  |  |  |  |  |  |  |  |  |  |  |  |  |  |  |  |  |  |  |  |  |  |  |  |  |  |  |  |  |  |  |  |  | **-** |  |  |
|  | Parent Responses |  |  |  |  |  |  |  |  |  |  |  |  |  |  |  |  |  |  |  |  | **/** |  |  |  |  |  |  |  |  |  |  |  |  |  |  |  |  |  |  |  |  |  |  |  |  |  |  |  |  |  |  |  |  |  |  |  |  |  | **/** |  |  |
|  | Family Relationships |  |  |  |  |  |  |  |  |  |  |  |  |  |  |  |  |  |  |  |  |  |  |  |  |  |  |  |  |  |  |  |  |  |  |  |  |  |  |  |  |  |  |  |  |  |  |  |  |  |  |  | **/** |  |  |  |  |  |  |  |  |  |
|  | Family - Harmony/Less Conflict |  |  |  |  |  |  |  |  |  |  |  |  |  |  |  |  |  |  |  |  |  |  |  |  |  |  |  |  |  |  |  |  |  |  | **+/** |  |  |  |  |  |  |  |  |  |  |  |  |  |  |  |  | **/** |  |  |  |  |  |  |  |  |  |
|  | Family - Cohesion |  |  |  |  |  |  |  |  |  |  |  |  |  |  |  |  |  |  |  |  |  |  |  | **/** |  |  |  |  |  |  |  |  |  |  |  |  |  |  |  |  |  |  |  |  |  |  |  |  |  |  |  | **-/** |  |  |  |  |  |  |  |  |  |
|  | Family - Activities |  |  |  |  |  |  |  |  |  |  |  |  |  |  |  |  |  |  |  |  |  |  |  | **-/** |  |  |  |  |  |  |  |  |  |  |  |  |  |  |  |  |  |  |  |  |  |  |  |  |  |  |  | **/** |  |  |  |  |  |  |  |  |  |
|  | Family - E, I, Int, & A |  |  |  |  |  |  |  |  |  |  |  |  |  |  |  |  |  |  |  |  |  |  |  |  |  |  |  |  |  |  |  |  |  |  |  |  |  |  |  |  |  |  |  |  |  |  |  |  |  |  |  | **-/** |  |  |  |  |  |  |  |  |  |
|  | Family - C, O, & M |  |  |  |  |  |  |  |  |  |  |  |  |  |  |  |  |  |  |  |  |  |  |  |  |  |  |  |  |  |  |  |  |  |  |  |  |  |  |  |  |  |  |  |  |  |  |  |  |  |  |  | **/** |  |  |  |  |  |  |  |  |  |
| Pain intensity; Pain Frequency; Pain Sensitivity; + = Positive relationship with greater pain; - = Negative relationship with greater pain; / = No relationship with pain; _ = Prognostic factor/Predictive of greater pain intensity; E = Expressiveness; I = Independence; Int = Intellectual Orientation; A = Achievement Orientation, C = Control, O = Organization; M = Moral Emphasis  Internalizing symptoms includes stress. Depression symptoms include low mood, hopelessness, variability in mood, and inability to attenuate negative mood. Daily interference includes unpleasantness, and limitations. School functioning includes attendance, participation, cognitive functioning, and schooling outside of the home. Social functioning includes adaptive social skills, activity, self-control, assertion, cooperation, empathy, competence, acceptance, communication, support, and fewer concerns. Coping - distraction includes cognitive and behavioral distraction. Internalizing symptoms (parents) includes anxiety and depression. Daily interference (parents) includes burden, hassle, time, and fewer uplifts. Parent responses include distracting, punishing, and solicitous. Some measures have been reversed to maintain consistency across constructs. | | | | | | | | | | | | | | | | | | | | | | | | | | | | | | | | | | | | | | | | | | | | | | | | | | | | | | | | | | | | | | |
